# Co-Sensitization Effects of Indoline and Carbazole Dyes in Solar Cells and Their Neutral–Anion Equilibrium in Solution

**DOI:** 10.3390/ma15217725

**Published:** 2022-11-02

**Authors:** Mateusz Gierszewski, Adam Glinka, Marcin Ziółek

**Affiliations:** Faculty of Physics, Adam Mickiewicz University, Uniwersytetu Poznańskiego 2, 61-614 Poznań, Poland

**Keywords:** dye-sensitized solar cells, co-sensitization, solvatochromic studies, neutral–anion equilibrium study

## Abstract

Co-sensitization of two or more light-absorbing compounds on a TiO_2_ surface has recently become one of the most successful strategies in the development of dye-sensitized solar cells (DSSCs). The specific structure of the dyes for DSSCs implies that they can partly exist in anionic forms in popular solvents used for sensitization. Our study concerns the above two issues being analyzed in detail using the example of the popular carbazole (MK2) and indoline (D205) dyes, studied by stationary absorption and emission, femtosecond transient absorption (in complete cells and in the solutions), current-voltage measurements, DFT and TD-DFT theoretical calculations. After the addition of D205 to DSSC with MK2, the fill factor of the cells was improved, and the electron recombination between TiO_2_ and the dyes was blocked (observed on sub-nanosecond time scales). Thus, the active co-adsorbent can take the role of the typically used passive additive, like chenodeoxycholic acid. Evidence of the concentration-dependent equilibrium between neutral and anionic forms of dyes with different lifetimes was found in acetonitrile solutions (the best for sensitization), while in ethanol solution the dominant form was the anion (worse for sensitization). Our findings should help in better understanding the operation and optimization of DSSC.

## 1. Introduction

Increasing consumption of electricity has engendered a search for novel, efficient and renewable energy sources. Dye-sensitized solar cells (DSSCs) are novel photovoltaic devices that have recently been intensively studied. The key elements of DSSC systems are: the electron-transport agent (a nanocrystalline semiconductor), light-absorber (dye) and hole-transport agent (redox couple in the electrolyte). Cell operation is accompanied by a number of desirable processes, including electron injection, charge transport and dye regeneration. Moreover, there are two major undesirable recombination processes. The first one is the recombination of injected electrons with the oxidized dye or with the redox couple, which worsens the operation of the solar cell [1,2]. Relaxation of the excited state of the dye through internal conversion is the other undesirable process in the operation of a solar cell. One strategy to improve the performance of DSSC systems and increase solar power conversion efficiency (PCE) is the use of two or more dyes for sensitization (co-sensitization) [3,4]. Typically, different dyes at the appropriate concentration ratio are dissolved in the same solvents. The co-sensitizers should improve the light harvesting. The structure of co-sensitizers should be complementary to prevent dye aggregation and competitive adsorption. One strategy in DSSC system design is to use one dye with a high molar absorption coefficient (ε) in the red range of the spectrum and another dye with a high ε of around 400 nm. This approach has often proven successful when an iodide electrolyte is used in the solar cells [5]. Co-sensitization may also reduce unwanted electron recombination between the electrons injected into the TiO_2_ film and the electrolyte due to the formation of a compacted molecular monolayer on the nanoparticle surface [6,7]. In addition, the appropriate size of the co-sensitizer molecule compared to that of the main dye is important. A mismatch may result in desorption of the main dye and finally a decrease in the efficiency of the solar cells [8]. Below, selected examples of the use of co-sensitization in DSSC systems with dyes belonging to different groups are presented. The use of eosin Y (a synthetic organic dye) and hibiscus sabdariffa (a natural dye) as a mixture of dyes to produce a DSSC cell has been reported to improve absorption in the range of 440–560 nm. In addition, higher photocurrent values were recorded compared to those in solar cells with a single dye. Electrochemical analyses indicated low charge-transfer resistance values for the eosin Y + hibiscus sabdariffa system, which also improved the performance of such solar cells [9]. Co-sensitization has proven to be a highly efficient approach for flexible DSSCs based on two metal-free dyes (JH-1 and SQ2). The long alkyl chain of JH-1 inhibited electron recombination, and the complementary spectral response in the co-sensitized device affected the efficiency of the entire device [10]. A positive effect of co-sensitization has been also demonstrated for DSSC systems in which one of the dyes was based on ruthenium [11,12,13,14,15,16,17,18,19,20]. The advantages of co-sensitization, such as improvement of optical absorption, IPCE response, electron lifetime, dye regeneration, dye coverage and cell preparation cost, have been demonstrated for DSSC cells sensitized with the dyes of the structures D-A-π-A and π-A in combination with a copper-based electrolyte [21]. Moreover, an increase in the open-circuit voltage due to a decrease in recombination between the electrons injected into TiO_2_ and a cobalt electrolyte has been reported for a DSSC cell using a pyridyl-based dye coded as T220 and co-sensitized with Dyenamo Blue dye [22].

Finally, all current top configurations of DSSCs (with the best laboratory photon conversion efficiency, PCE > 13% under 1 Sun, standard outdoor conditions) contain two types of dye absorbing in different spectral regions (with Cu-based electrolytes [23,24,25] or with Co-based electrolytes [26,27]). Moreover, the latest findings show that such DSSC devices can reach remarkable efficiencies of 34% [24] or 34.5% [25] under indoor illumination conditions, higher than most other photovoltaic technologies. This feature provides an opportunity for cheap DSSC power supply of low-energy-consuming indoor devices, targeted at the rapidly developing market of the Internet of Things (IoT) [24]. The key element in recent DSSC improvements is the use of novel copper- or cobalt-based electrolytes and a mixture of two dyes that are photoactive in different spectral regions.

Theoretical methods such as density functional theory (DFT) are helpful in the study of new dyes for potential application in DSSC systems and their adsorption on semiconductor surfaces [28,29]. In the case of a series of novel carbazole dyes, their polarizability made it possible to assess the anchoring properties of the dyes and the efficiency of electron injection into TiO_2_. Studies of the electronic structures permitted the assessment of the dyes’ capability of light collection. In general, the results of experimental studies have been satisfactorily supplemented by those of theoretical research [30].

**Figure 1:**
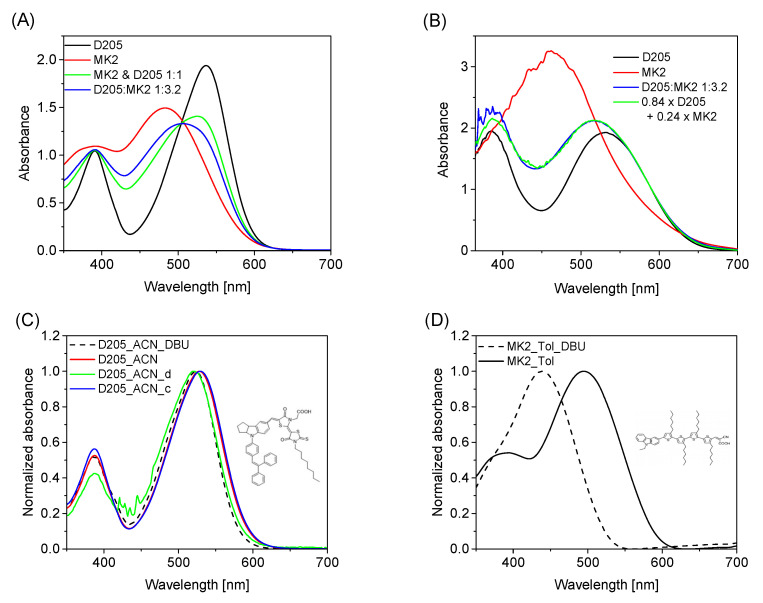
Stationary absorption of the solutions (ACN:Tol 1:1) used for co-sensitization (**A**), photoanodes after co-sensitization of MK2 and D205 (**B**), D205 (chemical structure as inset) in ACN with different dye concentrations and after addition of DBU (**C**), MK2 (chemical structure as inset) in Tol and after addition of DBU (**D**). The other absorption spectra of D205 are included in Figure S3.

Carbazole and indoline dyes are popular efficient dyes used in DSSCs. In particular, the best reported DSSC photon conversion efficiencies (PCE) for the two dyes investigated in this work—MK2, belonging to the carbazole derivatives [31], and D205, an indoline derivative—are 9.7% [32] and 9.5% [33], respectively. Figure S1 (and insets in Figure 1) shows the structures of both dyes. Solar cells with the above dyes (but not their mixture) have been recently extensively studied by us using time-resolved spectroscopic methods [34,35,36,37,38]. Due to the differences in the absorption spectra of both dyes, it is possible to improve the spectral response in the range of ~400 nm and ~500 nm, in which a lower absorption of the D205 dye is observed and, at the same time, the absorption maximum of MK2 is located. In addition, we demonstrate the formation of the anionic forms of the MK2 and D205 dyes in various organic solvents. The anionic form is formed by deprotonation of the carboxyl group, which is simultaneously the unit anchored to the TiO_2_ surface. Experimental studies were supplemented with the results of theoretical calculations using the DFT and TD-DFT methods, which revealed the difference in electronic structure between the anionic and neutral forms of the studied dyes.

To the best of our knowledge, there are no systematic studies in the literature of the co-sensitization of DSSC cells by these two popular dyes; in particular, no attempts at determination of charge separation dynamics on the ultrafast time scale have been reported. Moreover, the absorption bands of the D205 and MK2 mixture are quite complementary after sensitization on TiO_2_ (Figure 1). This ensures the collection of photons over a wider range of wavelengths. Moreover, the time constants of the complex of D205 and MK2 (in its neutral and anionic forms and at a neutral–anion equilibrium) as well as the deactivation processes upon excitation, depending on the organic solvent used, are discussed.

## 2. Materials and Methods

### 2.1. Preparation of the Solar Cells

The photoanodes: The photoanodes were prepared in a way similar to that described in our previously published articles [39,40]. Generally, glass plates were cut out from an FTO glass sheet (Sigma Aldrich, 2.2 mm thickness, 13 Ω/sq, Burlington, MA, USA) to a final size of 12 × 17 mm^2^. Next, they were cleaned using commercially available dishwashing detergent, distilled water and EtOH in an ultrasonic bath. Many configurations of cells with different films of TiO_2_ nanoparticles were deposited by the screen-printing or doctor-blade techniques on FTO glass plates and heated at 450 °C for 60 min. The resultant thickness of TiO_2_ films varied from 1.5 μm to 12 μm. SEM cross sections of TiO_2_ films were measured in our previously published articles, giving their thickness [41,42]. Details of the TiO_2_ paste used and film thickness are given in the Section 3 and in the legends to Tables S1–S6. For most DSSC configurations, pre- and post-TiCl_4_ treatment was applied, unless indicated otherwise in the tables.

The counter electrodes: Similarly, the counter electrodes were prepared according to the procedure described in our recently published articles [35,42]. One layer of activated platinum (Platisol T, Solaronix, Aubonne, Switzerland) was deposited on the cleaned surface of the electrodes. The photoanodes and counter electrodes were joined through a polymer seal (25 μm Surlyn, Meltronix, Solaronix SA) with the conducting surfaces facing inwards. Afterwards, the devices were filled with electrolyte through 1 mm holes in the counter electrode and sealed with a cover glass on the top.

The co-sensitization process: Two popular dyes belonging to the carbazole (MK2) and indoline (D205) groups were used to prepare the studied DSSC systems (both from Sigma-Aldrich). The glass plates with a mesoporous TiO_2_ layer were immersed in the dye solutions for about 16 h to ensure efficient adsorption of the dyes. The solutions of both dyes (D205: 0.14 mM and MK2: 0.21 mM) used for sensitization were dissolved in a 1:1 mixture of ACN and Tol. The concentration ratio of both dyes in solution in each case is specified in detail in the Section 3.

Electrolytes: Four types of electrolytes were used for cell preparation. Their specifications can be found in the manuscript and in the legends of Tables or Figures. The compositions and concentrations of individual components are given below. 

The first of them, “Co-Bpy”, was a cobalt-based electrolyte made of the following components: 0.25 M [Co(bpy)_3_][B(CN)_4_]_2_, 0.035 M [Co(bpy)_3_][B(CN)_4_]_3_, 0.1 M LiClO_4_ and 0.5 M tert-butylpyridine (TBP) dissolved in ACN. The redox couple was a cobalt–bipyridine complex (Co-Bpy). 

The second one, “Co-Phen”, was a cobalt-based electrolyte made of the following components: 0.25 M [Co(phen)_3_](TFSI)_2_, 0.035 M [Co(phen)_3_](TFSI)_3_, 0.1 M LiTFSI and 0.5 M tert-butylpyridine (TBP) dissolved in ACN. The redox couple was a cobalt–phenanthroline complex (Co-Phen). 

The third one, “iodide with 0.5 TBP”, was an iodide-based electrolyte made of the following components: 0.08 M I_2_, 0.6 M 1,2-dimethyl-3-propylimidazolium iodide (DMPII), 0.1 M LiI, 0.1 M guanidine thiocyanate (GuSCN) and 0.5 M TBP dissolved in ACN.

The fourth one, “iodide without TBP”, was an iodide-based electrolyte made of the following components: 0.08 M I_2_, 0.6 M 1,2-dimethyl-3-propylimidazolium iodide (DMPII), 0.1 M LiI and 0.1 M guanidine thiocyanate (GuSCN) dissolved in ACN.

### 2.2. MK2 and D205 Solutions Studies

The MK2 and D205 dyes have been studied in solvents with different polarity and protic-aprotic properties. For D205, the following solvents were used in the studies: acetonitrile (ACN), dichloromethane (DCM), ethanol (EtOH), toluene (Tol), tert-butanol (tert-Bu) and tetrahydrofuran (THF). The MK2 dye has been studied in Tol. For both dyes in each organic solvent, the anionic form was generated using DBU: 1,8-diazabicyclo [5.4.0]undec-7-ene. The used dyes, solvents and DBU are commercially available (from Sigma-Aldrich). The MK2 and D205 solutions were prepared at three different concentrations. In the manuscript, the label “_c” stands for concentrated [c ~ 10**^−^**^4^ M], while “_d” stands for diluted [c ~ 10**^−^**^6^ M]. The symbol of the dye and solvent without additional description means that the concentration of the dye in the solution is moderate [c ~ 10**^−^**^5^ M]. The anionic form was generated using DBU in a solution with a moderate dye concentration [c ~ 10**^−^**^5^ M].

A Hitachi F-7000 fluorescence spectrometer was used to measure the stationary fluorescence spectra of MK2 and D205 in the solutions. Fluorescence quantum yields were determined using rhodamine 6G in water as a standard, with $\Phi_{F}^{st}$ = 0.90 [43]. The values of fluorescence quantum yields for D205 dye in different solvents were calculated according to the equation below:

(1) $$\Phi_{F}=\Phi_{F}^{st}\frac{\int F_{X}(1-10^{-A_{st}})}{\int F_{st}(1-10^{-A_{X}})}\frac{{\left(n_{X}\right)}^{2}}{{\left(n_{st}\right)}^{2}}$$

Here, ∫*F_X_* is the area under the emission spectrum of the studied sample, ∫*F_st_* is the area under the emission spectrum of the standard, *A_X_* and *A_ST_* are the absorbance of the studied sample and standard at an excitation wavelength, respectively, *n_X_* is the solvent refractive index of the studied sample, *n_st_* is the solvent refractive index of the standard, and $\Phi_{F}^{st}$ is the value of the fluorescence quantum yield of the standard.

More technical aspects about DSSC characterization, stationary and transient absorption studies of complete solar cells as well as the MK2 and D205 dyes in the solutions are included in the SI.

## 3. Results and Discussion

### 3.1. Co-Sensitization of the Dyes in the Cells

We investigated the effect of co-adsorption of MK2 and D205 dyes on TiO_2_ by stationary absorption measurements of the photoanodes, current–voltage and IPCE measurements of the complete cells and femtosecond transient absorption measurements of whole cells treated as samples. Different ratios of MK2:D205 concentration, different types of TiO_2_ layers and different electrolytes (iodide- and cobalt-based) were tested. No common anti-aggregation additives (like chenodeoxycholic acid, CDCA) were used for sensitization in order to clearly observe the effects of the interaction of the two dyes in the mixtures.

Figure 1A,B presents examples of sensitization effects on the absorption properties of a relatively thin (2.5 μm) layer of TiO_2_ at a D205:MK2 concentration ratio of 1:3.2 in the solution. As can be seen in Figure 1A, the minimum D205 absorption in this solution is around 450 nm, while in this spectral region the absorption of the MK2 dye is close to the maximum. The complementary absorption spectra of the two dyes were one of the reasons for choosing them for co-sensitization studies. The absorption spectra recorded on TiO_2_ (Figure 1B) show some important differences with respect to those in solution. First of all, the absorption onset of both dyes in solution is finished at around 650 nm (Figure 1A), while those on TiO_2_ extend above 700 nm for D205 and even above 750 nm for MK2 (Figure 1B). The difference is even more pronounced in the IPCE spectra, shown in Figure S2 (in the Supplementary Materials).

Secondly, unlike in the sensitizing solutions, the maximum absorbance of MK2 alone on TiO_2_ is almost twice that of D205. Taking into account the about 1.5 times-smaller extinction coefficient of MK2 compared to D205 (in Tol: 38400 M^−1^cm^−1^ vs. 59300 M^−1^cm^−1^, according to [31] and our current measurements, respectively), this implies that about 3 times more MK2 than D205 molecules are adsorbed on the same TiO_2_ layer (absorbance ratio × extinction coefficient ratio: 2 × 1.5). Higher dye loading of MK2 can probably be explained by the dye structures (shown in Figure S1 and in insets in Figure 1C,D). The structure of MK2 is more “linear”, with the −COOH anchoring unit at one end, while in D205 the anchoring unit is in the middle of the dye structure; therefore, D205 cannot be as densely packed on TiO_2_ nanoparticles as MK2.

Interestingly, D205 dye is adsorbed much faster on TiO_2_ than MK2 molecules. This is probably due to the difference in the values of the acid dissociation constant between the MK2 and D205 anchoring groups. Therefore, thirdly, the dye ratio on TiO_2_ is much different from that in solution, in favor of D205. As can be seen in Figure 1B, the absorption spectra of the dyes’ mixture on TiO_2_ (1:3.2 sensitizing D205:MK2 ratio) are much more similar to the spectra of D205. The mixture absorbance on TiO_2_ can be fitted by adding 0.84 times the contribution of D205 absorbance and 0.24 times the MK2 absorbance (Figure 1B), which, taking into account different extinction coefficients, corresponds to a ratio of 69% to 31% of D205 and MK2 molecules, respectively, on TiO_2_. Table 1 summarizes the fitted contributions of absorbance of both dyes and different ratios of molecules on TiO_2_ for different initial concentration ratios in solution.

Next, the solar cells were prepared in different configurations, and their photovoltaic parameters were compared. Special attention was paid to the character of changes in the parameters of the cells with the dye mixtures, relative to those in the cells with one type of dye. Table 2 shows the exemplary parameters measured for the as-produced cells (measured 60 min after preparation): a 12 μm thick TiO_2_ layer composed of mixtures of nanoparticles with thicknesses of 18 nm and 30 nm and larger scattering nanoparticles and filled with Co-Bpy electrolyte. The highest short-circuit current density (J_SC_) was found for the cells with MK2. However, the cells with a mixture of D205 and MK2 exhibited a much higher fill factor (FF); therefore, their overall PCE was slightly higher than that of MK2 cells.

The parameters for the six other configurations are summarized in Tables S1–S6 (in Supplementary Information). The maximum relative errors (standard deviation/average value) of the photovoltaic parameters for the screen-printed cells were 1% for V_OC_ and 3% for FF, J_SC_ and PCE, while for doctor blade cells they were: 1% for V_OC_, 6% for FF and J_SC_ and 10% for PCE. Particular photovoltaic parameters in each configuration depend on many factors that are well known from previous DSSC studies. For example, the open circuit voltage (V_OC_) increases in different electrolytes in the order: I^−^/I_3_^−^ (Tables S1 and S2), Co-Bpy (Table 2, Tables S5 and S6) and Co-Phen (Tables S3 and S4), in accordance with the more positive redox potential of the redox pair. Except for the iodide configuration without TBP, the J_SC_ of the cells with only D205 is significantly lower than that of those with only MK2, and the relative photocurrent (total_APCE [34]) is also lower, indicating poorer efficiency of the absorbed photons’ conversion. The FF of D205 cells was always better than that of MK2 cells (probably due to the differences in molecular structures and better blocking of the TiO_2_ surface from contact with redox pairs in the cells with adsorbed D205). Increasing the thickness of TiO_2_ layer results in higher J_SC_ and higher PCE values, but the total_APCE becomes lower (Tables S2–S6). However, what is most important is that the trends indicated in Table 1 were confirmed for all other configurations in Tables S1–S6: the use of a mixture of the dyes always results in the improvement of the J_SC_ with respect to the cells with D205, and in the improvement of FF with respect to the cells with MK2. In other words, the addition of MK2 to the cells with D205 does not affect their high FF, but it increases significantly their J_SC_. On average, the photocurrent of the co-adsorbed mixtures is higher by 25 ± 8% (relative to that of D205 cells, standard error of the mean indicated), while the improvement in the fill factor is 10 ± 2% (relative to that of MK2 cells). The latter result is especially important because it proves that co-adsorbed active molecules can produce the same effect as commonly used passive anti-aggregation additives (e.g., CDCA). Such additives occupy an area on TiO_2_ surface but, unlike the co-adsorbed sensitizing dyes, do not contribute to light absorption and electron injection into TiO_2_.

The overall PCE of the cells with the dye mixtures is sometimes higher and sometimes lower than that of MK2 cells, depending on how much D205 cells show poorer performance than MK2 cells in a particular configuration. For example, for pure anatase TiO_2_ (paste from Dynamo) and TBP in solution, the resulting shift of the TiO_2_ conduction band edge (towards more negative potentials) reduces the driving force for electron injection, and the J_SC_ of the cells with D205 is then drastically lower than that of MK2 cells (Tables S3 and S4). It should also be noted that D205 cells quickly lose their PCE due to the drop of the photocurrent on the time scale of hours, while MK2 cells keep their PCE constant or sometimes even improve their J_SC_, probably due to the irradiation effects studied by us recently [44]. For the cells with the D205:MK2 mixtures, a decrease in performance was also observed (but not as much as for the cells with D205 alone); therefore, after 4 h or more, the PCE of the MK2 cells was always the highest. However, their FF was always lower than that of the D205:MK2 cells.

**Figure 2:**
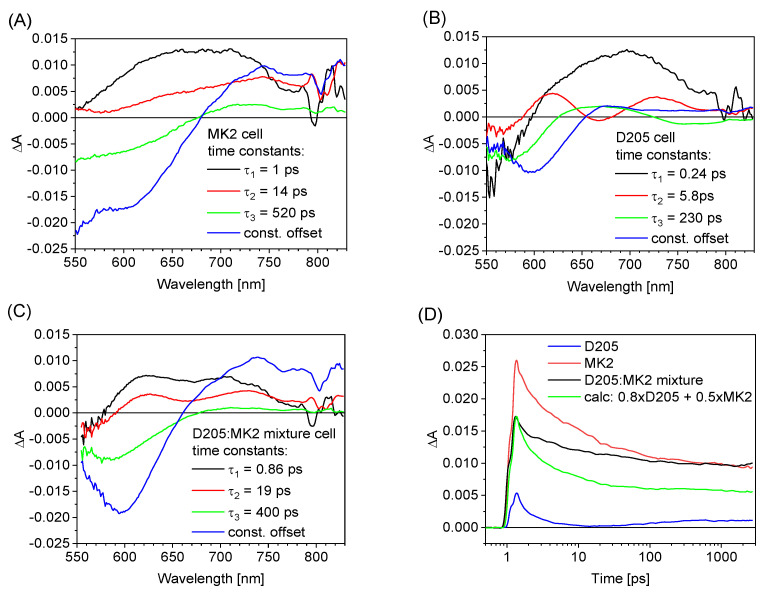
The wavelength-dependent amplitudes of the fitted components (3-exponential fit with a constant offset) and the corresponding time constants of the exemplary cells with only MK2 (**A**), only D205 (**B**) and the dyes mixture (**C**). The transient absorption kinetics at 750 nm for the studied samples (**D**).

In this section we will also present the results of femtosecond transient absorption studies of the cells with the dye mixture. The transient absorption of the cells was measured upon excitation at 470 nm and globally analyzed in a spectral range from 560 to 830 nm and in a temporal window of up to 3 ns. Figure 2A,C shows the wavelength-dependent amplitudes of the fitted components (3-exponential fit with constant offset) and the corresponding time constants of the exemplary cells with only D205, only MK2 and their mixture, respectively. The relative error of the given time constants is less than 10%. The interpretation of the results for the cells with single dyes is performed along the same lines as in our earlier studies of indoline [37,38,45] and carbazole dyes [34,35,39,40]; therefore, it will be only briefly summarized here. In the cells with MK2 only (Figure 2A), the electron injection takes place on a fast time scale, within the first component (1 ps) and partially within the second component (14 ps). The third component of the fit for MK2 cells (520 ps) has the same spectral shape as that of the constant offset component; therefore, it is assigned to the partial recombination of the injected electrons from TiO_2_ to the oxidized dye. The fastest component for D205 (Figure 2B) of a time constant close to that of the IRF (0.24 ps) is due to the decay of the initially excited (Franck–Condon) state (also called the locally excited, or LE, state). The second (6 ps) and the third (230 ps) time constants were assigned to the decay of the subsequent charge transfer (CT) state of the indoline dye (hot and cold), respectively. Its decay is due to electron injection and undesired self-quenching of CT states. The residual spectrum (constant offset component) is proportional to the successfully separated charges and contains the contributions of the ground state depopulation (bleach, negative signal below 650 nm) and the absorption of oxidized dye and electrons into TiO_2_ (positive signal above 650 nm).

The results for the cells with the dye mixture (Figure 2C) contain the contributions from both the D205 and MK2 cells. The most important finding can be deduced from the constant offset component, which is dominated by the signal of MK2 (compare Figure 2A,B). However, in contrast to the cell with only MK2, the intercept of the residual spectrum with the zero-absorption axis is shifted to shorter wavelength (around 660 nm for the cell with the mixture instead of 680 nm for that with MK2) and the contribution of the constant offset component is greater. Such changes are characteristic of the MK2 cells upon addition of CDCA, as observed by us earlier. In the cells with MK2 and CDCA, the ratio of the residual to the initial signal amplitude at 750 nm (close to the absorption maximum of the oxidized dye) was significantly higher than that for the cell without CDCA due to the suppression of electron recombination [35]. A similar tendency can be clearly observed when monitoring the transient absorption kinetics at 750 nm for the samples studied, which are shown in Figure 2D. As can be seen, the residual signal at 3 ns for the cell with the dye mixture (black line) is almost twice as high as that calculated from the individual contributions from the cells with D205 or MK2 (green line), which should be explained by a higher relative population of the charge-separated species at 3 ns and a smaller contribution of fast electron recombination. Along with the above-mentioned increased fill factor of the cells with the dye mixture (with respect to that of MK2 cells), this is more evidence that the co-adsorbed active molecule (D205) can play a similar role in blocking electron recombination as the CDCA additive. We have also tried to obtain additional information by analyzing the time evolution of the transient absorption signals. Figure S4 (in Supplementary Materials) shows the transient absorption spectra of D205, MK2 and co-sensitized cells by D205 and MK2 at selected time delays, as well as the dye mixture spectrum obtained by adding the individual contributions of D205 and MK2 best fitting the above spectra. The contributions from individual dyes change from 0.8 × D205 + 0.55 × MK2 at 0.6 ps to 0.7 × D205 + 0.7 × MK2 at 3 ns. This means that the contribution of the MK2 signal increases slightly over time with respect to that of D205, which could be an indication of partial electron transfer from the excited state of D205 to the excited state of MK2. Such a process is indeed possible, as the LUMO energy of D205 is slightly higher than that of MK2 (potentials −0.91 V vs. SHE [34] and −0.89 V vs. SHE [31], respectively). However, such a hypothesis requires verification with further evidence.

### 3.2. Solvatochromic Studies and Anionic Forms of the Dyes

The stationary absorption spectra of D205 in ACN at different dye concentrations and after DBU addition (to create the anionic form: D205^−^) and of MK2 in Tol, are included in Figure 1C,D. The positions of the longest-wavelength absorption maximum for both dyes, under different conditions, are given in Table 3. In general, the position of the absorption maximum of D205 is influenced by the type of solvent used and often changes after DBU addition. Taking into account the two solvent parameters [46]: α (the ability of the solvent to donate a hydrogen bond) and β (the ability of the solvent to accept hydrogen bond), it seems that the β parameter has a greater impact on the position of the absorption maximum of D205 in a given solvent. The longest-wavelength absorption maximum was found for DCM (546 nm at β = 0.10), while the shortest-wavelength one was for EtOH (527 nm at β = 0.75). Similar conclusions were formulated on the basis of D205 solvatochromic studies with more solvents used; in the solvents with lower H-bond ability, a shift of the absorption maximum to the longer wavelengths was observed [47]. Given the structure of the D205 molecule, it seems most likely that a hydrogen bond is formed between a hydrogen atom from the carboxyl group of D205 and a molecule of a given solvent. A change in the concentration of the dye may result in a significant or only slight shift of the absorption maximum, which is largely dependent on the solvent used. Taking into account the change in the concentration of D205 in ACN from ~10*^−^*^4^ M (ACN_c) through 10*^−^*^5^ M (ACN) to 10*^−^*^6^ M (ACN_d), it can be concluded that the blue shift of the absorption maximum (from 528 nm to 519 nm) is observed as a result of dilution of the solution (Figure 1C and Table 3). By diluting the D205 solution in ACN, the formation of D205^−^ is preferred, similar to TPC1 dye [48]. This is confirmed by the impact of using DBU. The addition of this organic base to a solution of D205 in ACN with a concentration of ~10*^−^*^5^ M results in the formation of D205^−^, accompanied by a shift of the absorption maximum from 528 nm to 523 nm. A pronounced blue shift after DBU addition was found in DCM, Tol and THF solutions. Moreover, the results obtained for D205 in DCM at concentrations in the range of ~10*^−^*^4^ M to ~10*^−^*^6^ M indicate that upon dilution of the solution, the absorption maximum is at the same wavelength (546 nm, spectra not shown in the manuscript). In the case of DCM, Tol and THF, it can be postulated that regardless of the dye concentration, the neutral species of the dye predominates. A completely different picture emerges when EtOH is used as a solvent of D205. The addition of DBU does not cause a significant shift of the absorption maximum (527 nm in EtOH and 528 nm after DBU addition). This may indicate the dominance of the anionic form of D205 in the pure EtOH. This is consistent with the hypothesis presented in the literature, which indicates that the anionic form of similar indoline dyes is favored in alcohols and ACN, while in alkyl halides (DCM) the neutral form of the dye predominates [47,49]. For the MK2 dye in Tol, after the DBU addition, a clear blue shift in the absorption maximum from 495 nm to 439 nm (Table 3) was observed. A similarly large change in the absorption maximum position has been found for TPC1 (453 nm in DCM and 406 nm in DCM + DBU) [48]. In addition, a large positive Mulliken charge (0.43) for the hydrogen atom in cyanoacrylic acid has been confirmed [48]. Hence, it seems that for polar solvents with a high β parameter, including alcohols and ACN, the −COOH group is more easily deprotonated, and the anionic form of the dye is favored, unlike in solvents with low values of β (DCM, Tol). Much smaller changes in absorption upon the transition from the neutral to anionic form of D205 (~10 nm), compared to those noted for MK2 and TPC1 (~50 nm), indicate increased acidity of the hydrogen atom in the carboxyl group of D205 dye; therefore, it deprotonates more easily, and the dye attaches to the TiO_2_ surface faster than MK2.

Next, we wanted to find out the impacts of the type of solvent used and DBU addition on the chemisorption of the D205 and MK2 dyes on the TiO_2_ surface. The absorption spectra of D205 and MK2 from the solutions in DCM, ACN and EtOH, sensitized on the TiO_2_ surface along with those recorded after the addition of DBU, are presented in Figure S5. The same time of sensitization and the same D205 concentration (~10*^−^*^5^ M) were applied. The greatest absorbance of the photoanodes was found after sensitization of D205 in ACN; absorbance was slightly lower in DCM, and it was the least for D205 dye from EtOH attached to TiO_2._ This may indicate a beneficial effect of the equilibrium between the anionic and neutral forms of the dye on sensitization. The use of DBU in any case prevents effective chemisorption of D205 on the TiO_2_ surface. A similar effect was observed for MK2 in Tol after the DBU addition. This is probably due to the interaction of the cation formed from the DBU with the TiO_2_ surface.

**Figure 3:**
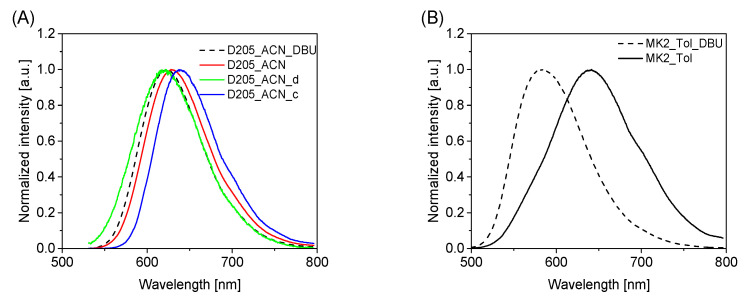
Fluorescence spectra of D205: (**A**) in ACN at different concentrations or with addition of DBU, (**B**) MK2 in Tol solution or with addition of DBU; λ_exc_ = 480 nm (MK2) or 525 nm (D205). Fluorescence spectra of D205 in the other organic solvents considered are included in Figure S6.

Further confirmation of different anion–neutral form equilibria in different solvents comes from steady-state fluorescence and time-resolved studies. The fluorescence spectra of D205 (Figure 3A and Figure S6) in different organic solvents are significantly affected by DBU addition and the type of organic solvent used. In general, a blue shift of the fluorescence maximum was found in all solvents due to DBU addition (Table 3). Only for EtOH does DBU addition not cause any changes in the position of the fluorescence maximum (627 nm). A distinct blue shift is observed for D205 in ACN in concentrations changed from 10^−4^ M through 10^−5^ M to 10^−6^ M (637 nm, 628 nm and 621 nm, respectively). The value observed for the most diluted dye solution is the closest to that observed upon DBU addition to the D205 solution in ACN (623 nm). A clearly different result was found for D205 in Tol, for which the fluorescence maximum was located at a much shorter wavelength than in the other solvents (584 nm) and after DBU addition (571 nm). This may be related to the long-lived LE state in this solvent (instead of the CT state in the other solvents), which was confirmed by the transient absorption measurements (see below). An even more pronounced blue shift of the fluorescence maximum after DBU addition was found for MK2 in Tol (from 642 nm to 582 nm, Figure 3B and Table 3).

The excited-state lifetimes of D205/D205^−^ and MK2/MK2^−^ are presented in Table 3 and were determined from the femtosecond transient absorption signal probed at 730 nm (for MK2 and D205 in Tol) or at 600 nm (for D205 in the other, more polar solvents). As reported before [38,39] and also confirmed below, 730 nm corresponds to the maximum of the transient absorption signal of the excited state of MK2 and the LE state of D205, while 600 nm corresponds to the transient absorption maximum of the CT sate of D205 in polar solvents. Examples of the transient absorption kinetics at 600 nm for D205 in ACN solutions at different dye concentrations and after DBU addition are included in Figure S7. The excited state lifetimes change with D205 concentration in ACN (from 350 ps at ~10^−4^ M to 480 ps at ~10^−5^ M and finally to 520 ps at ~10^−6^ M). After DBU addition to the solution, the excited-state lifetime is similar to that of ~10^−6^ M without DBU. This confirms again the concentration dependence of the D205/D205^−^ equilibrium in ACN and the domination of the anionic form of D205 only in the diluted D205 solution in ACN. In contrast, almost no changes in lifetime were found in EtOH after DBU addition, confirming that the anionic form dominates at all dye concentrations. For both D205 and MK2 dyes, the fluorescence quantum yield (Φ_F_) and radiative rate constant (k_r_) values are slightly higher for the anionic form than for the neutral one (Table 3). Moreover, Φ_F_ and k_r_ are also significantly higher for the LE state (D205 in Tol) than for the CT state (D205 in the other solvents studied), indicating the possible twisted character of the CT state. The CT-state lifetimes of the neutral and anionic forms of D205 show different dependence on the solvent parameters. The lifetime of D205^−^ increases with increasing β and decreases with increasing α and solvent polarity. In contrast, for the neutral form, the CT lifetime depends mainly on the polarity and is significantly reduced in the most polar ACN solvent.

Representative transient absorption spectra of D205 in DCM and after DBU addition and those of MK2 in Tol and after DBU addition, for selected time delays between the pump and pulse probe, are presented in Figures S8 and S9, respectively. Transient absorption studies with the use of single solvents have been presented in our previously published articles for D149/D205 [34,38,50] and MK2 [35,39]. The new contribution in our present study is the use of DBU, which induces the transition to the anionic form. At this point, we want to draw attention to the comparison between the transient absorption spectra of D205 in DCM (only neutral form) and the same solution after DBU addition (only anionic form). After excitation, the main positive bands, with maxima at ~720 nm and between ~1400 nm and ~1500 nm, were observed for D205_DCM and D205_DBU_DCM. Their positions can be assigned to the initially locally excited (LE) state of the neutral and anionic forms of D205. The negative feature of the spectrum, with a maximum at ~540 nm, is due to the ground state depopulation, while that at ~650 nm is due to the stimulated emission. On the sub-ps and single ps time scales, further evolution of transient absorption spectra was observed, with a red shift of the stimulated emission and changes in the shape of the positive signals (in VIS and NIR). Both for D205_DCM and D205_DBU_DCM, the transient absorption maxima were observed at ~600 nm and ~1400 nm, which can be attributed to the relaxed charge-transfer (CT) state. Generally, the transient absorption spectra of the neutral and anionic forms of D205 are quite similar. For D205, the transient absorption of CT state above 700 nm (in the range of stimulated emission) contains a more negative contribution from the neutral than from the anionic form. For MK2, the difference in the position of the negative stimulated emission band can be observed in the VIS range (more long-wavelength for the neutral form than for the anionic form), while the transient absorption amplitude in the band tail above 1000 nm in the NIR range was higher for the neutral from than for the anionic form.

**Figure 4:**
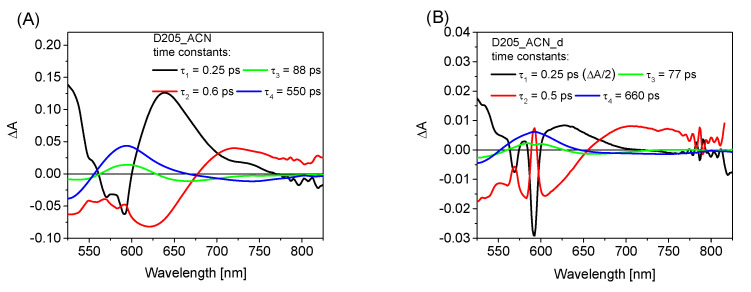

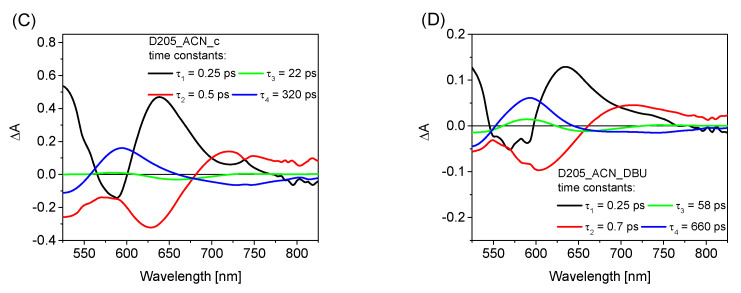
The wavelength-dependent amplitudes of the fitted components (4-exponential fit with the fixed fastest component) and the corresponding time constants of D205 in ACN at different concentrations (**A**–**C**) and after DBU addition (**D**). The fastest component (0.25 ps, close to the IRF) was fixed during the fit. The spikes below 600 nm observed in the fastest components and most pronounced for the diluted samples are due to the Raman signals from pure solvents.

To acquire more information about the solvation times and excited-state lifetimes, a global analysis of transient absorption spectra was performed. Figure 4 shows the wavelength-dependent amplitudes of the fitted four exponential components and the corresponding time constants for D205 in ACN at different concentrations and after DBU addition, both in the VIS and NIR ranges. The results for the other solvents are given in Figures S10–S15. The interpretation of the time constants follows that presented in our previous papers [34,38,50]. In all solvents studied except for Tol, the two fastest components of the wavelength-dependent amplitudes are due to the ultrafast decay of the LE state and solvation dynamics. Solvation times increase in this order, from the shortest to the longest: ACN (below 1 ps), DCM, THF, EtOH and finally tert-Bu. In the latter solvent, the remarkably slow solvation with the longest component of >100 ps (which influences the third component in the global analysis) should be emphasized. The longest components represent the decay of the CT state (LE in the case of Tol). Their dependence on D205 concentration and DBU addition is fully consistent with the findings from the single-wavelength kinetics (in Table 3). For ACN (Figure 4) the lifetime increases from that for the most concentrated sample (dominated by the neutral form) to that of the most diluted one, which shows the same lifetime as for D205^−^ (the samples with DBU). For DCM (Figure S11), whose neutral form is present for all D205 concentrations, the lifetimes are the same and significantly longer than upon addition of DBU. For EtOH with the domination of D205^−^, the lifetimes with DBU and without it are the same (Figure S12). Interestingly, the concentration-dependent measurements in tert-Bu reveal that the CT lifetime slightly increases upon dilution up to the values obtained for D205_tert-Bu_DBU (Figure S15). This indicates that, despite having a higher β parameter (0.94 in tert-Bu vs. 0.75 in EtOH) not all dyes exist in the anionic form at moderate concentrations in tert-Bu. Most probably, the anionic form is additionally stabilized by the higher polarity of the solvent because its dielectric constant is significantly higher in EtOH (24.5) than in tert-Bu (10.9).

A conclusion following from this section is that the spectroscopic studies of D205 and D205 + DBU (a strong organic base) in solution have revealed the existence of an equilibrium between the neutral and anionic forms of D205 in different solvents. D205^−^ has slightly blue-shifted (~10 nm) absorption and emission maxima with respect to those of the neutral form. D205 exists mainly in the neutral form in toluene, tetrahydrofuran and dichloromethane solvents, while in ethanol (having strong polarity and proton-accepting properties), it exists mainly in the anionic form. In acetonitrile (having only weak proton-accepting properties) and tert-butanol (of low polarity), a concentration-dependent equilibrium between D205 and D205^−^ is observed, with more anions in more-diluted solutions. The changes in the absorption position as a function of the solvent polarity and the differences between neutral and anionic forms have been confirmed by TD-DFT calculations. The domination of anions in the D205 sensitizing solution was found to have a negative influence on the dye’s adsorption ability on a mesoporous TiO_2_ electrode. The highest dye loading was observed to take place from the acetonitrile solution; that from the ethanol solution was almost twice as low, while no adsorption was observed from the solutions containing D205 + DBU.

Femtosecond transient absorption studies in solution yielded information about the characteristic time constants of the complex D205 deactivation processes upon excitation. In non-polar toluene, the LE state decays with an average time of 200 ps (similar for neutral and anionic forms). In polar solvents, the subsequent CT state is formed within solvation times that vary: 0.5 ps in acetonitrile, 1.5 ps in dichloromethane, 3 ps in tetrahydrofuran, 30 ps in ethanol and up to >100 ps in tert-butanol. The radiative rate constant of the LE state is about 4 times higher than that of the CT state. The lifetime of the CT state of D205^−^ decreases in more-protic solvents (from 500–600 ps in dichloromethane and acetonitrile to 350 ps in ethanol), while the lifetime of the CT state of D205 neutral form decreases with increasing solvent polarity (from 700 ps in dichloromethane to 350 ps in acetonitrile).

### 3.3. Theoretical Studies and Anionic Forms of the Dyes

To elicit more information about the electronic structures of D205 and MK2 in their neutral (D205/MK2) and anionic (D205^−^/MK2^−^) forms in the ground and excited states, density functional theory (DFT) and time-dependent DFT (TD-DFT) calculations were performed. Geometries of D205 in a vacuum, D205*/*D205^−^ using a CPCM solvation model with EtOH, DCM, ACN and Tol as solvents were optimized using the b3lyp functional together with a 6–31G(d) basis set in the Gaussian 09 software [51]. This theoretical method was previously used for the characterization of D149 and D205 dyes (D149 belongs to the same family of indoline dyes and has a similar structure to D205) [36,52,53,54,55,56]. The geometry of MK2 molecules was optimized in the vacuum using four different theoretical methods: b3lyp/6–31G(d), b3lyp/6–31G(d,p), pbe1pbe/6–31G(d) and cam-b3lyp/6–31G(d).

**Figure 5:**
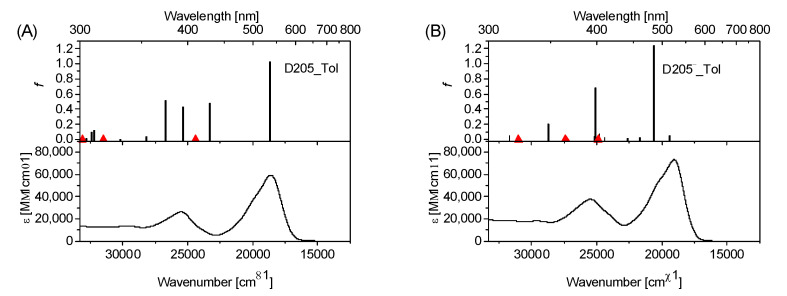
Absorption spectra of D205 (neutral, (**A**)) and D205^−^ (anion, (**B**)) in Tol. Predicted transition energies and oscillator strengths *f* are indicated by solid vertical lines. The (prohibited) transitions involving the n,π* singlet states are marked by red triangles. The results obtained for D205 in the other organic solvents are included in Figure S16. All theoretical results were determined at the b3lyp/6–31G(d) level of theory.

Figure 5, Figures S17 and S18 show the absorption spectra of D205, D205^−^, MK2 and MK2^−^ in different organic solvents (D205, D205^−^ in ACN, EtOH, DCM and Tol; MK2, MK2^−^ in Tol). The experimental absorption spectra were compared to the theoretical results of the predicted transition energies and oscillator strengths f. The theoretical results were determined at the b3lyp/6–31G(d) level of theory. Tables S7–S9 include the calculated [b3lyp/6–31G(d)] singlet excitation energies with the corresponding oscillator strengths, *f*, starting from the optimized geometry of the ground states of D205/D205^−^ and MK2/MK2^−^, using the CPCM solvation model with ACN, EtOH, DCM (D205) and Tol (D205 and MK2) as solvents. In general, a satisfactory correspondence between the results of experimental stationary absorption and theoretical calculations is observed for both the neutral and anionic forms of both studied dyes. A common feature of both dyes in their neutral and anionic forms is the π,π* character of S_0_→S_1_ transition. As follows from a comparison of the theoretically determined S_0_→S_1_ transition energy with the longest-wavelength absorption band of D205, in the polar solvents (ACN, EtOH), the calculated energy is red-shifted with respect to the absorption maximum in a given solvent. This trend is more pronounced for the neutral form of D205 (~800 cm*^−^*^1^) compared to D205^−^ (~300 cm*^−^*^1^). However, in the non-polar or low-polar solvents (Tol, DCM), the opposite tendency is noted, with differences in the range from ~100 cm*^−^*^1^ to ~400 cm*^−^*^1^. A comparison of the calculated energy of S_0_→S_1_ electronic transitions between D205 and D205^−^, in the same solvent, indicates a clear blue shift of the maximum for the anionic form. The results are consistent with those obtained for D149 dye [49]. Higher energy transitions for D205 and D205^−^, regardless of the solvent used in the CPCM model (S_0_→S_2_ and subsequent), mostly show the π,π* character. Some π,π* transitions are accompanied by n,π* transitions with low oscillator strength. A comparison of the determined transition energies S_0_→S_1_ and S_0_→S_2_ indicates significant differences between D205 and D205^−^, regardless of the solvent used. For D205, ΔE ≈ 5000 cm*^−^*^1^, while for D205^−^ it is in the range from ~1000 cm*^−^*^1^ to 2600 cm*^−^*^1^. This is primarily due to the marked shift of the energy of the S_0_→S_2_ transition to lower values for the anionic form compared to that of the neutral form.

For the neutral form of MK2 in the gas phase, DFT and TD-DFT calculations were carried out using different functionals (b3lyp, pbe1pbe and cam-b3lyp) in combination with two basis sets: 6–31G(d) and 6–31G(d,p). The results are shown in Table S7. Regardless of the calculation method used, the S_0_→S_1_ transition had the π,π* character. Maintaining the same basis set, 6–31G(d), but changing the functional from b3lyp to pbe1pbe results in a clear red shift (to 544.8 nm); however, the use of the cam-b3lyp functional induces a clear blue shift of the S_0_→S_1_ maximum (to 388.8 nm). The use of a slightly larger basis set in 6–31G(d,p) instead of 6–31G(d) has a rather slight impact on the energy levels of the HOMO and LUMO, as well as on the S_0_→S_1_ electronic transition for MK2 in the gas phase. A comparison of the results obtained for MK2 within the CPCM solvation model with Tol (λ = 491.1 nm) and with the experimental absorption spectrum in this solvent (λ = 495 nm) implies that the DFT and TD-DFT calculations for MK2 reflect the experimental results in the best way when using the b3lyp functional.

Figure S18 shows the shape of the frontier molecular orbitals of D205 and D205^−^ obtained within the CPCM solvation model with ACN as a solvent at the b3lyp/6–31G(d) level of theory. Generally, due to the deprotonation of the carboxyl group in D205, the distributions and locations of the HOMO and LUMO do not change significantly. The HOMO of D205 and D205^−^ is located mainly on the indoline part of the molecules. This agrees with the results obtained for D149 [52] and D205 in the gas phase [34]. The LUMO of D205 and D205^−^ is located mainly on the rhodamine part of the molecules. The S_0_→S_1_ electronic transition between HOMO and LUMO can be considered as an intramolecular charge-transfer transition due to a significant separation between the HOMO and the LUMO distributions.

The shapes of the HOMO and the LUMO of MK2 in its neutral (MK2) and anionic (MK2^−^) forms, determined at the b3lyp/6–31G(d) theory level using the CPCM solvation model with Tol as a solvent, are shown in Figure S19. The S_0_→S_1_ electronic transition for both forms of MK2 takes place to a large extent between these orbitals. In the case of MK2, the distributions and locations of the HOMO and LUMO are distinctly different. The HOMO is evenly located on the π-conjugated system (oligothiophenes), in part also on carbazole, which is an electron donor group. On the other hand, its LUMO is clearly concentrated near the cyanoacrylic acid moiety, which is the dye’s anchoring group to the nanoparticle surface. For MK2^−^, the HOMO is practically not found within the carbazole part. Most of this orbital is located on the oligothiophenes and on the cyanoacrylic acid anchoring group. In addition, its LUMO is largely located on oligothiophene units and to a small extent on the anchoring part.

## 4. Conclusions

Co-sensitization of popular and efficient carbazole (MK2) and indoline (D205) dyes, which have complementary absorption spectra, has been tested in DSSCs. A significantly greater amount of MK2 than D205 dye in solution (>4:1) is necessary to obtain similar numbers of molecules of both dyes on a TiO_2_ surface. The cells with MK2 alone show a better J_SC_ but a lower FF than those with only D205 in the same configuration. The addition of MK2 to the cells with D205 results in maintaining their high FF while significantly increasing their J_SC_ (by about 25%). The increase in the FF of the cells with the dye mixture with respect to that of MK2 alone (by about 10%) means that D205 dye can passivate the TiO_2_ surface to curb unwanted electron recombination between TiO_2_ and the electrolyte, which so far has usually been realized by anti-aggregation additives like CDCA that do not absorb light.

Further evidence of positive co-adsorption effects (similar to those of CDCA addition) was found by femtosecond transient absorption studies of complete cells. The residual signal at about 3 ns after excitation was significantly higher in the cells with the D205:MK2 mixture than that expected from the individual contributions of both dyes based on the results obtained for the cells with only D205 or only MK2. This indicates that the initial charge separation is more efficient in the cells with the dye mixture and that electron recombination between TiO_2_ and oxidized dyes is suppressed.

On average, the overall PCE of the mixture of D205 and MK2 is not yet significantly higher than that of the cells with MK2 dye alone, but this is probably due to a too-low electron injection efficiency of D205, which is lower than that of MK2. The PCE improvement should be stronger if dyes of more comparable relative photocurrents are used. Nevertheless, our results give more insight into the benefits of the recent superior strategy of using two or more dyes in top-efficiency DSSCs. The active dyes can block the electron recombination of each other and, at the same time, contribute to light harvesting and electron injection. In this way, the effective light harvesting can be realized with a thinner TiO_2_ layer, which is especially important in cells with cobalt- or copper-based electrolytes in modern DSSCs. The contribution of the cascade process of electron injection (from the excited state of one dye to the excited state of another one, and then to TiO_2_) cannot be also excluded.

Both dyes were also studied in solution. D205 exists mainly in the neutral form in toluene, tetrahydrofuran and dichloromethane solvents. Evidence of the concentration-dependent equilibrium between the neutral and anionic forms of dyes with different lifetimes was found in acetonitrile and tert-butanol solutions, while in ethanol solution the dominant form was the anion. The domination of anions in the D205 sensitizing solution has a negative influence on the dye-adsorption ability of a mesoporous TiO_2_ electrode. The excited-state lifetimes of D205 lie in the range of electron injection times observed for D205 in DSSCs (longer component of 230 ps), which might limit the electron injection quantum yield. In contrast, for MK2 dyes with a much higher relative photocurrent (total_APCE parameter) than that of D205, the electron injection times in DSSCs (1 ps and 14 ps) are significantly shorter than those in solution (550 ps in toluene).

## Figures and Tables

**Table 1:** The fitted contributions of absorbance of MK2 and D205 dyes and different ratios of molecules on TiO_2_ for different initial concentration ratios in solution.

D205:MK2 Concentration Ratio in Solution	D205:MK2 Absorbance Ratio on TiO_2_	Number of Dyes (Normalized to Only D205 Sample)	D205:MK2 Ratio of Dyes Concentrations on TiO_2_
1:0 (only D205)	1.00:0.00	1.00	100%:0%
1:1.5	0.92:0.16	1.17	79%:21%
1:2	0.86:0.16	1.11	78%:22%
1:3.2	0.84:0.24	1.21	69%:31%
1:4.2	0.90:0.40	1.52	59%:41%
0:1 (only MK2)	0.00:1.00	3.08	0%:100%

**Table 2:** Photovoltaic parameters of DSSCs sensitized by D205 or MK2 separately and by a mixture D205:MK2 1:4.2 (Co-Bpy electrolyte, TiO_2_ layer: 12 μm, paste 18 + 30 NR + scattering, Dyesol).

Sample	V_OC_, V	FF	J_SC_, mA/cm^2^	PCE, %
D205	0.72	0.66	8.95	4.24
MK2	0.74	0.57	11.54	4.82
D205:MK2 1:4.2	0.72	0.66	11.32	5.41

**Table 3:** Spectral and photophysical results for D205 and MK2 dyes in different organic solvents.

Solution	λ_abs_ [nm]	λ_F_ [nm]	Փ_F_	τ [ps]	*k*_r_/10^8^ [s^−1^]	Σ*k*_nr_/10^8^ [s^−1^]
D205						
ACN_c	528	637	0.03	350	0.9	27.7
ACN	528	628	0.05	480	1.1	19.7
ACN_d	519	621	0.07	520	1.3	17.9
ACN_DBU	523	623	0.08	540	1.4	17.1
DCM	546	632	0.07	670	1.0	13.9
DCM_DBU	536	622	0.08	500	1.6	18.4
EtOH	527	627	0.05	350	1.4	27.2
EtOH_DBU	528	627	0.05	330	1.4	28.9
Tol	537	584	0.10	190 *	5.1	47.6
Tol_DBU	524	571	0.10	130 *	7.7	69.2
tert-Bu	533 **	618	0.09	600	1.5	15.2
tert-Bu_DBU	531	615	0.15	830	1.8	10.2
THF	532	626	0.05	550	0.9	17.3
THF_DBU	524	622	0.07	850	0.8	11.0
MK2						
Tol	495	642	0.15	670 *	2.2	12.7
Tol_DBU	439	582	0.21	570 *	3.7	13.9

λ_abs_ is the position of the lowest-energy bands in the absorption spectra, λ_F_ is the fluorescence emission maximum, Փ_F_ is the fluorescence quantum yield, τ is the excited-state lifetime (from femtosecond transient absorption measurements at λ = 600 nm or * 730 nm), *k*_r_ is the radiative rate constant, and Σ*k*_nr_ is the sum of non-radiative rate constants; moreover: _c (c ~ 10^−4^ M), _d (c ~ 10^−6^ M), _DBU (with 1,8-diazabicyclo [5.4.0]undec-7-ene), ** 536 nm for concentrated D205 solution in tert-Bu (10^−4^ M).

## Data Availability

Not applicable.

## Supplementary Materials

The following are available online at https://www.mdpi.com/article/10.3390/ma15217725/s1, Figure S1: Scheme of D205 (left) and MK2 (right) dyes. Table S1: Photovoltaic parameters for the cells: TiO_2_ layer from doctor blade with P25 paste, no TiCl_4_ treatment, electrolyte: iodide with 0.5 TBP. Table S2: Photovoltaic parameters for the cells: TiO_2_ layer 2.5 μm from screen-printed 30TS paste, Dyenamo, no TiCl_4_ treatment, electrolyte: iodide without TBP. Table S3: Photovoltaic parameters for the cells: TiO_2_ layer 2.5 μm from screen-printed 30 TS paste, Dyenamo, electrolyte Co-Phen, the best cell. Table S4: Photovoltaic parameters for the cells: TiO_2_ layer 1.5 μm from screen-printed diluted 30 TS paste, Dyenamo, electrolyte Co-Phen, the best cell. Table S5: Photovoltaic parameters for the cells: TiO_2_ layer 12 μm from screen-printed 18 NRT, Dyesol, electrolyte Co-Bpy, the best cell. Table S6: Photovoltaic parameters for the cells: TiO_2_ layer 6 μm from screen-printed 30 NRD, Dyesol, electrolyte Co-Bpy, the best cell. Figure S2: Exemplary IPCE spectra of pure D205, pure MK2 and a mixture of the two dyes. The cell configuration corresponds to that of Table 2 (A) and Table S1 (B). Figure S3: Absorption spectra of D205: (A) in DCM at different concentrations or with addition of DBU, (B) in different organic solvents (c ~ 10−5 M) with or without addition of DBU. Figure S4: Transient absorption spectra at a given time delay between pump and probe pulse of D205 and MK2 and their mixtures. Figure S5: Absorption spectra of D205 from the solutions in DCM, ACN and EtOH, sensitized on the TiO_2_ surface along with those recorded after addition of DBU (A); The absorption spectra of MK2 from the solutions in Tol, sensitized on the TiO_2_ surface along with those recorded after addition of DBU (B). Figure S6: Fluorescence spectra of D205 in different organic solvents (c ~ 10−5 M) with or without addition of DBU; λexc = 525 nm. Figure S7: Transient absorption kinetics at 600 nm for D205 in ACN solution at different dye concentrations and after DBU addition. Dashed line: 2-exp fit of the selected kinetics. The lifetime given in Table 3 is calculated as the amplitude-weighted average of both components. Figure S8: Representative transient absorption spectra for selected time delays between pump and probe pulse of D205 in DCM solution and after DBU addition (A,C—VIS; B,D—NIR). Figure S9: Representative transient absorption spectra for selected time delays between pump and probe pulse of MK2 in Tol solution and after DBU addition (A,C—VIS; B,D—NIR). Figure S10: The wavelength-dependent amplitudes of the fitted components and the corresponding time constants of the D205 in ACN at 10−4 M (A) and DBU addition (B) in NIR range. Figure S11: The wavelength-dependent amplitudes of the fitted components (4-exponential fit) and the corresponding time constants of the D205 in DCM and after DBU addition (A,C: VIS, B,D: NIR). Figure S12: The wavelength-dependent amplitudes of the fitted components (4-exponential fit) and the corresponding time constants of the D205 in EtOH and after DBU addition (A,C: VIS, B,D: NIR). Figure S13: The wavelength-dependent amplitudes of the fitted components and the corresponding time constants of the D205 in THF and after DBU addition (A,C: VIS, B,D: NIR). Figure S14: The wavelength-dependent amplitudes of the fitted components (4-exponential fit with the fitted fastest component) and the corresponding time constants of the D205 in Tol and after DBU addition (A,C: VIS, B,D: NIR). Figure S15: The wavelength-dependent amplitudes of the fitted components (4-exponential fit with the fitted fastest component) and the corresponding time constants of the D205 in tert-Bu at different dye concentrations and after DBU addition (A–D: VIS, E: NIR). Table S7: Theoretical results obtained for D205 and D205− using CPCM solvation model with EtOH, ACN, DCM and Tol as solvents. Results for MK2 in vacuum using different theory levels and the CPCM solvation model with Tol as a solvent (MK2 and MK2−). Table S8: Calculated [b3lyp/6–31G(d)] singlet excitation energies with the corresponding oscillator strengths *f* starting from optimized geometry of the ground states of D205 and MK2 using CPCM solvation model with ACN, EtOH, DCM (D205) and Tol (D205 and MK2) as solvents. Table S9: Calculated [b3lyp/6–31G(d)] singlet excitation energies with the corresponding oscillator strengths *f* starting from optimized geometry of the ground states of D205− and MK2− using CPCM solvation model with ACN, EtOH, DCM (D205) and Tol (D205 and MK2) as solvents. Figure S16: Absorption spectra of D205 (neutral; A, C, E) and D205− (anion; B, D, F) in EtOH (A,B), DCM (C,D) and ACN (E,F). Predicted transition energies and oscillator strengths *f* are indicated by solid vertical lines. The (prohibited) transitions involving the n,π* singlet stares are marked by red triangles. All theoretical results were determined at the b3lyp/6–31G(d) level of theory. Figure S17: Absorption spectra of MK2 (neutral; A) and MK2− (anion; B) in Tol. Predicted transition energies and oscillator strengths f are indicated by solid vertical lines. The (prohibited) transitions involving the n, π* singlet stares are marked by red triangles. All theoretical results were determined at the b3lyp/6–31G(d) level of theory. Figure S18: The shape of the HOMO and the LUMO of D205_ACN and D205−_ACN at the b3lyp/6–31G(d) theory level responsible for the S0→S1 electronic transition determined as isodensity surface plots. Figure S19: The shape of the HOMO and the LUMO of MK2_Tol and MK2−_Tol at the b3lyp/6–31G(d) theory level responsible for the S0→S1 electronic transition determined as isodensity surface plots.
