# Supplementary material for: Co-Sensitization Effects of Indoline and Carbazole Dyes in Solar Cells and Their Neutral–Anion Equilibrium in Solution
Source: Materials (Basel). 2022 Nov 2;15(21):7725. doi: 10.3390/ma15217725 (PMC9657344; doi:10.3390/ma15217725)
Supplement: Supplementary file 1 [file materials-15-07725-s001.zip › materials-1973168-supplementary.pdf]

Supplementary Materials

Co-Sensitization Effects of Indoline and Carbazole Dyes in Solar Cells and Their Neutral–Anion Equilibrium in Solution

Mateusz Gierszewski *, Adam Glinka and Marcin Ziółek *

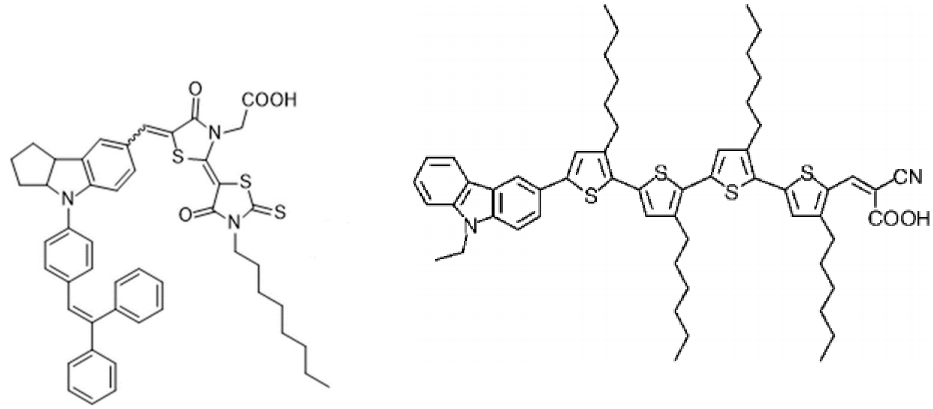

Figure S1. Scheme of D205 (left) and MK2 (right) dyes.

Table S1. Photovoltaic parameters for the cells: TiO_2 layer from doctor blade with P25 paste, no $TiCl_4$ treatment, electrolyte: iodide with 0.5 TBP.

Sample	V_{OC}, V	FF	J_{SC}, mA/cm²	PCE, %	Total_APCE
D205	0.67	0.58	5.87	2.30	0.42
MK2	0.70	0.52	7.21	2.64	0.49
D205:MK2 1:1.5	0.69	0.52	8.27	2.96	0.55
D205:MK2 1:3.2	0.69	0.57	8.79	3.43	0.51

Table S2. Photovoltaic parameters for the cells: TiO_2 layer 2.5 μm from screen-printed 30TS paste, Dyenamo, no $TiCl_4$ treatment, electrolyte: iodide without TBP.

Sample	V_{OC}, V	FF	J_{SC}, mA/cm²	PCE, %	Total_APCE
D205	0.68	0.54	9.01	3.29	0.75
MK2	0.64	0.43	9.83	2.70	0.79
D205:MK2 1:1.5	0.67	0.51	9.02	3.06	0.69
D205:MK2 1:3.2	0.65	0.47	9.01	2.78	0.69

Table S3. Photovoltaic parameters for the cells: TiO_2 layer 2.5 μm from screen-printed 30TS paste, Dyenamo, electrolyte Co-Phen, the best cell.

Sample	V_{OC}, V	FF	J_{SC}, mA/cm²	PCE, %	Total_APCE
D205	0.82	0.75	3.67	2.24	0.33
MK2	0.84	0.63	8.03	4.30	0.69
D205:MK2 1:2	0.82	0.75	4.26	2.64	0.37

Table S4. Photovoltaic parameters for the cells: TiO_2 layer 1.5 μm from screen-printed diluted 30TS paste, Dyenamo, electrolyte Co-Phen, the best cell.

Sample	V_{OC}, V	FF	J_{SC}, mA/cm²	PCE, %	Total_APCE
D205	0.84	0.76	3.61	2.3	0.41
MK2	0.88	0.68	7.44	4.44	0.70
D205:MK2 1:2	0.84	0.74	4.18	2.59	0.40

Table S5. Photovoltaic parameters for the cells: TiO_2 layer 12 μm from screen-printed 18NRT, Dyesol, electrolyte Co-Bpy, the best cell.

Sample	V_{OC}, V	FF	J_{SC}, mA/cm²	PCE, %	Total_APCE
D205	0.76	0.75	5.22	2.97	0.32
MK2	0.75	0.71	5.96	3.14	0.35
D205:MK2 1:4.2	0.74	0.75	6.78	3.76	0.41

Table S6. Photovoltaic parameters for the cells: TiO_2 layer 6 μm from screen-printed 30NRD, Dyesol, electrolyte Co-Bpy, the best cell.

Sample	V_{OC}, V	FF	J_{SC}, mA/cm²	PCE, %	Total_APCE
D205	0.75	0.67	5.88	2.95	0.41
MK2	0.79	0.68	9.53	5.16	0.68
D205:MK2 1:4.2	0.77	0.71	8.33	4.58	0.55

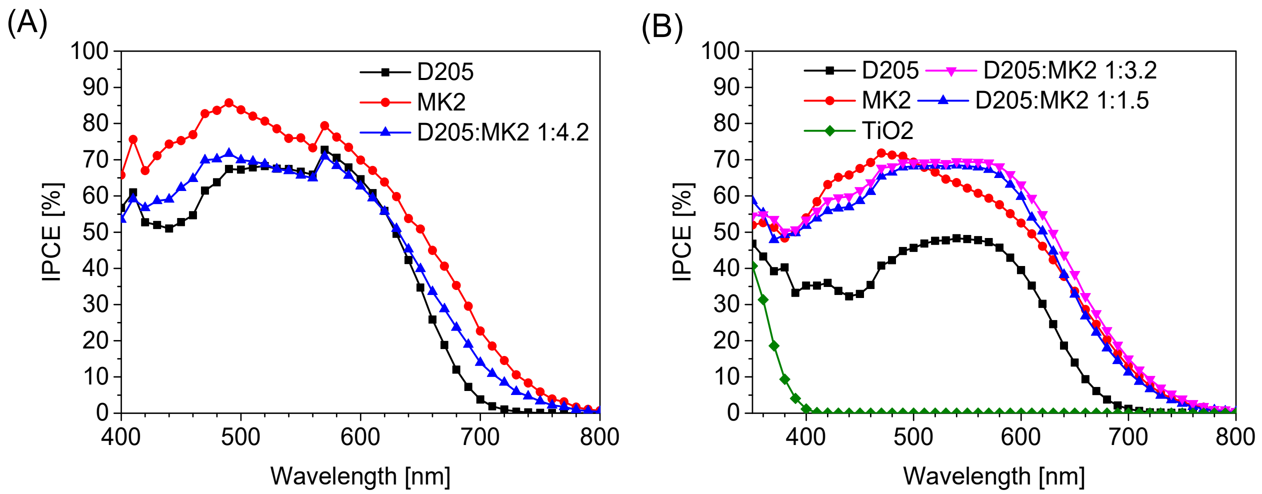

Figure S2. Exemplary IPCE spectra of pure D205, pure MK2 and a mixture of the two dyes. The cell configuration corresponds to that of Table 2 (A) and Table S1 (B).

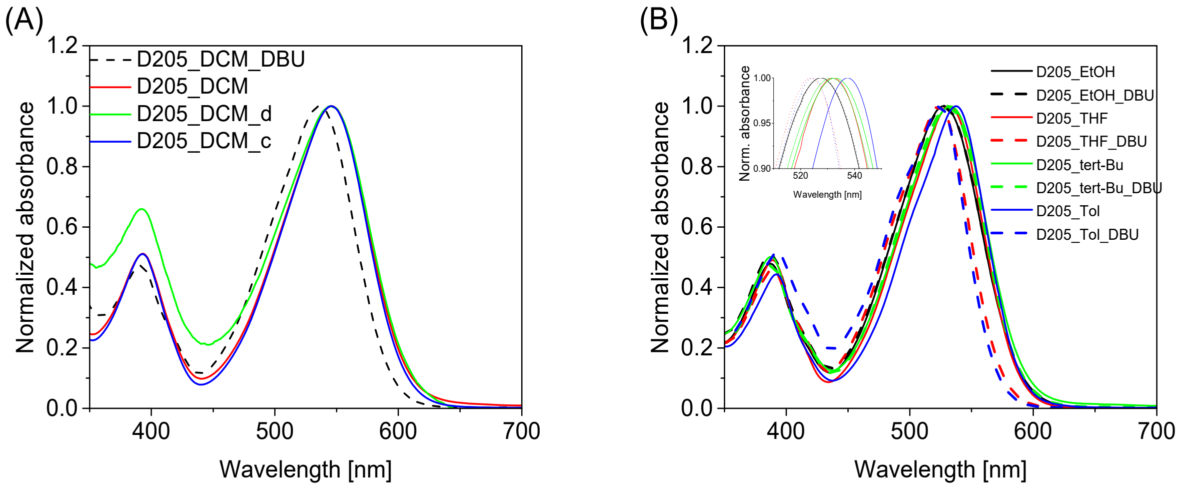

Figure S3. Absorption spectra of D205: (**A**) in DCM at different concentrations or with addition of DBU, (**B**) in different organic solvents (c ~ 10^{-5} M) with or without addition of DBU.

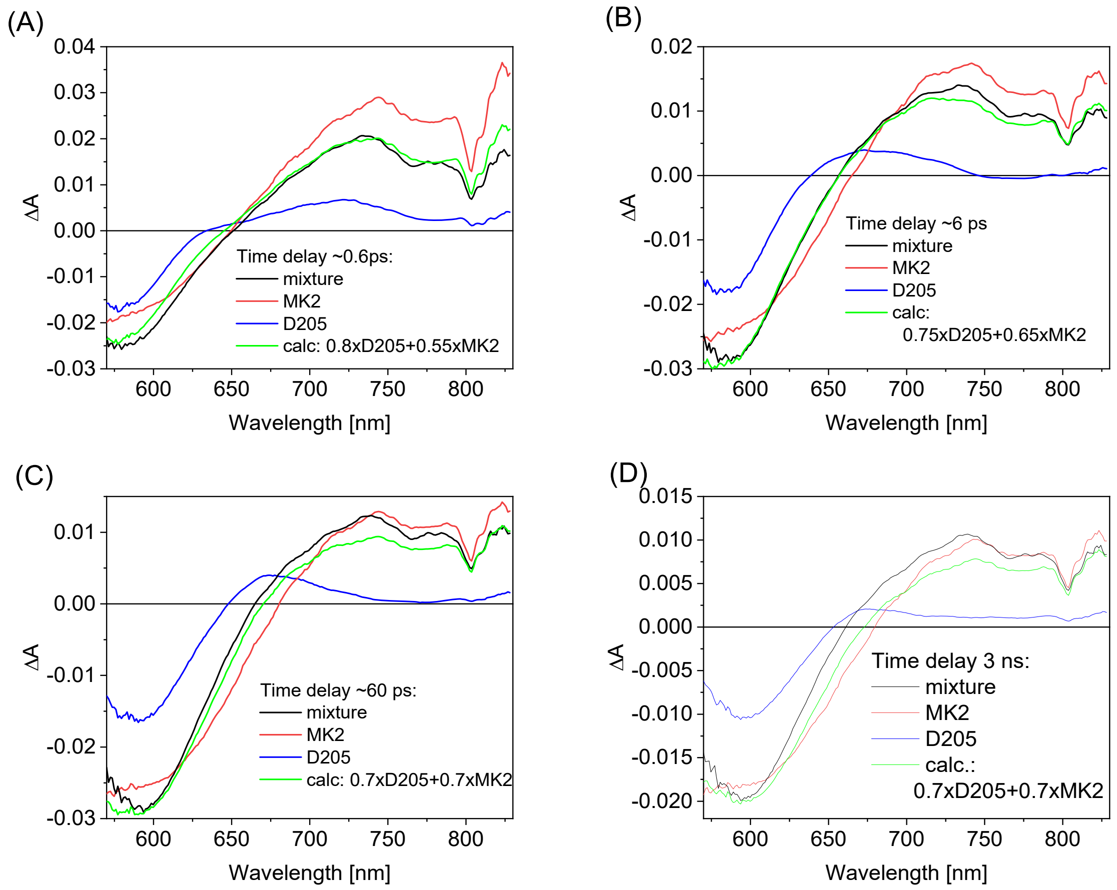

Figure S4. Transient absorption spectra at a given time delay between pump and probe pulse of D205 and MK2 and their mixtures.

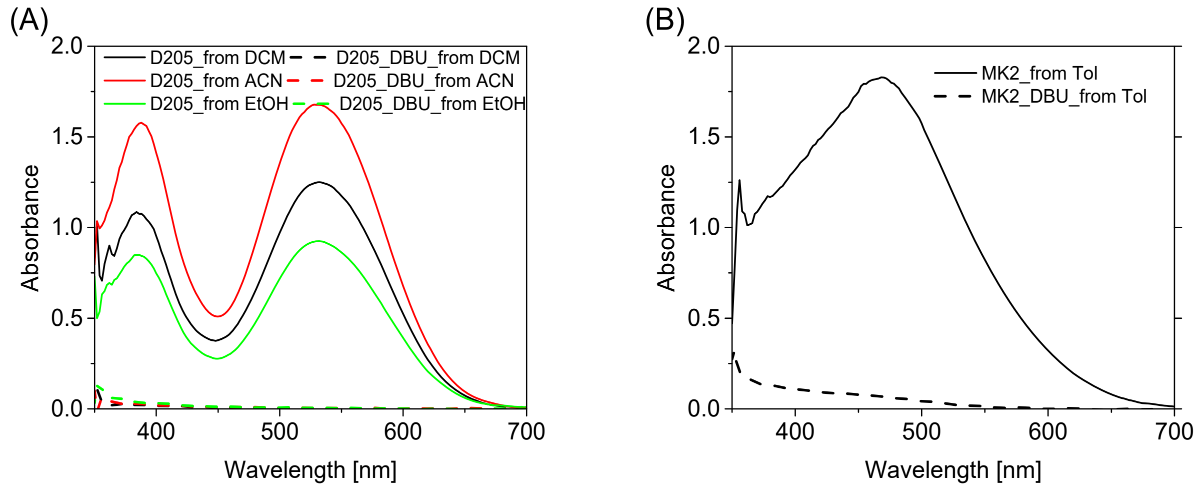

Figure S5. Absorption spectra of D205 from the solutions in DCM, ACN and EtOH, sensitized on the TiO_2 surface along with those recorded after addition of DBU (**A**); the absorption spectra of MK2 from the solutions in Tol, sensitized on the TiO_2 surface along with those recorded after the addition of DBU (**B**).

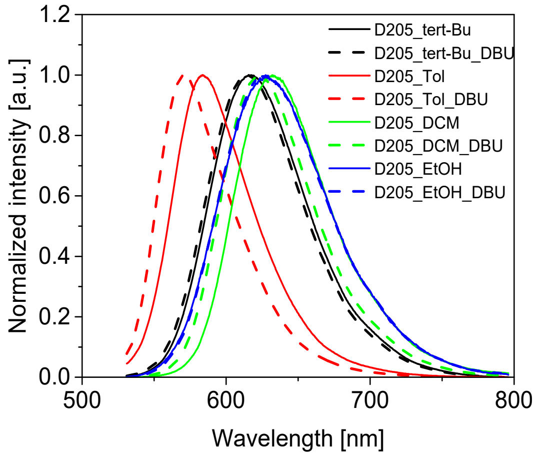

Figure S6. Fluorescence spectra of D205 in different organic solvents (c ~ 10^{-5} M) with or without addition of DBU; λ_{exc} = 525 nm.

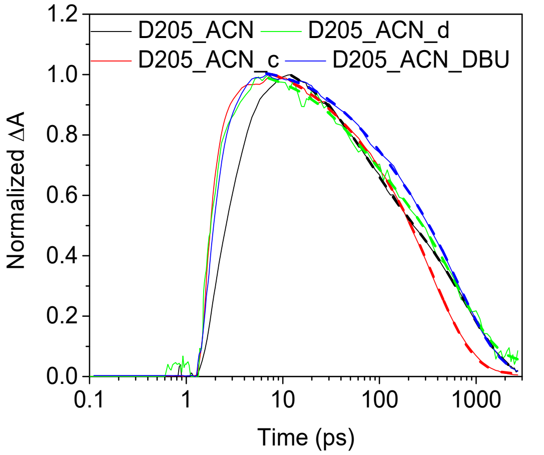

Figure S7. Transient absorption kinetics at 600 nm for D205 in ACN solution at the different dye concentrations and after DBU addition. Dashed line: 2-exp fit of the selected kinetics. The lifetime given in Table 3 is calculated as the amplitude-weighted average of both components.

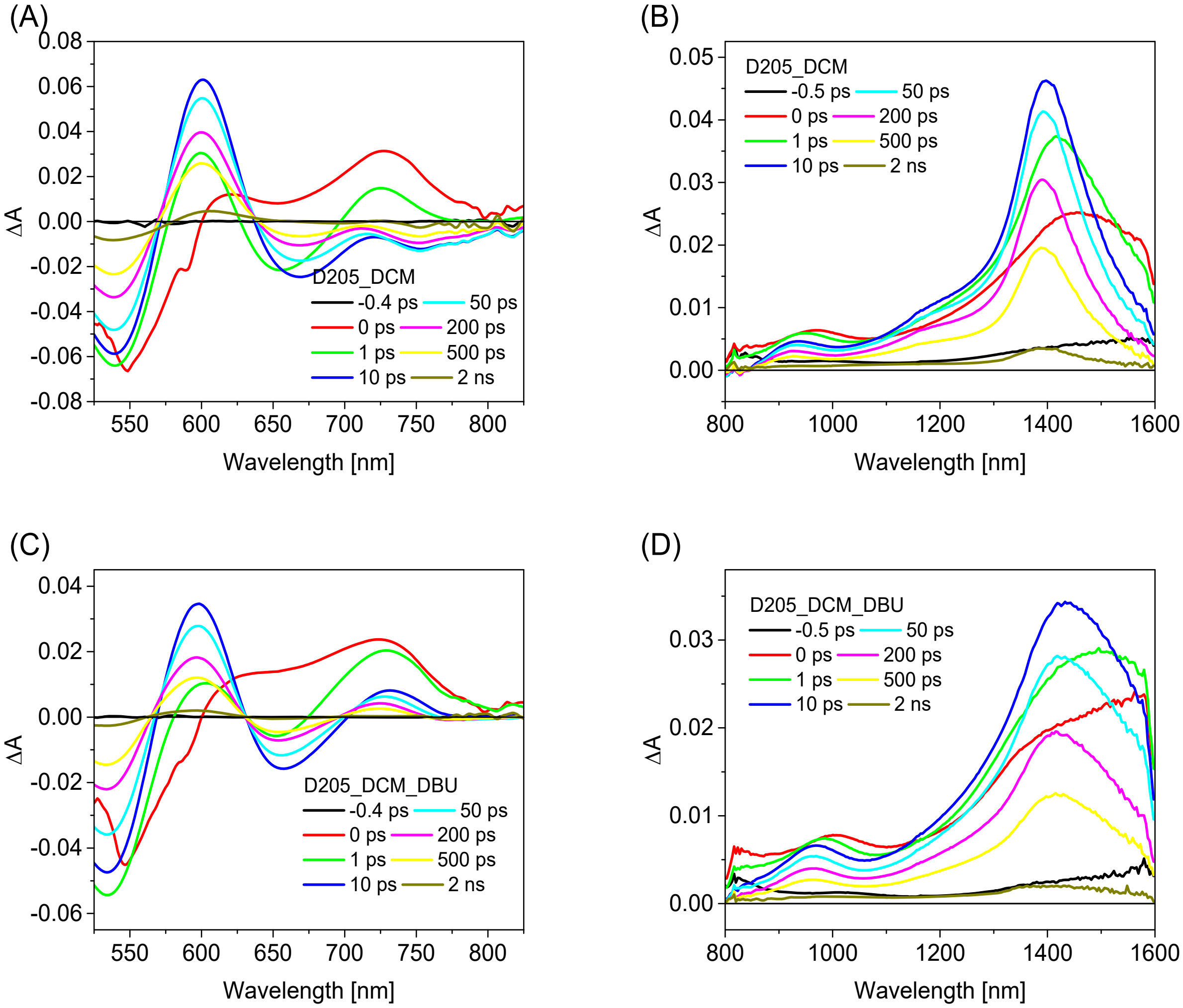

Figure S8. Representative transient absorption spectra for selected time delays between pump and probe pulse of D205 in DCM solution and after DBU addition (**A,C** – VIS; **B,D** – NIR).

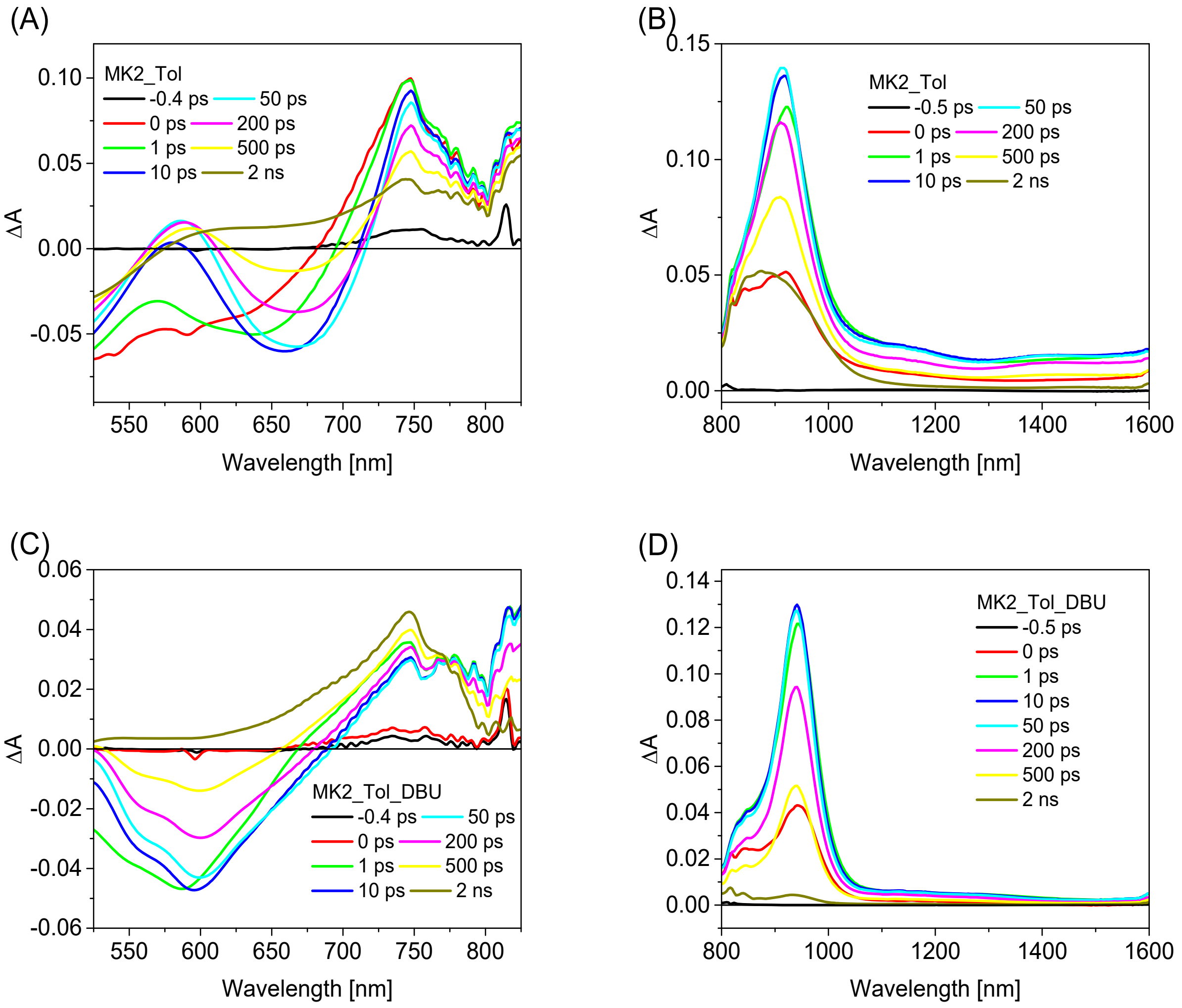

Figure S9. Representative transient absorption spectra for selected time delays between pump and probe pulse of MK2 in Tol solution and after DBU addition (**A,C** – VIS; **B,D** – NIR).

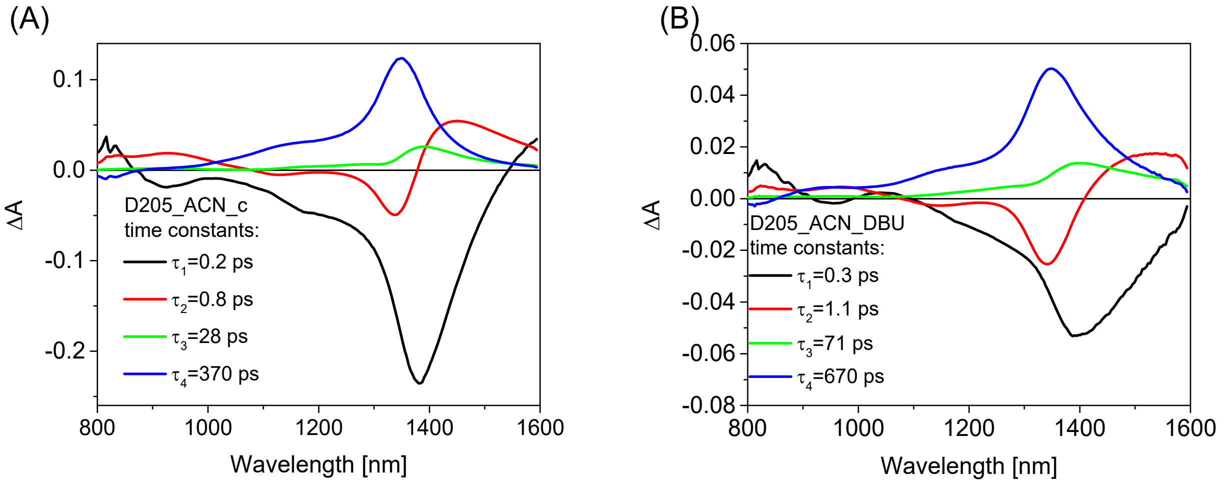

Figure S10. The wavelength-dependent amplitudes of the fitted components and the corresponding time constants of the D205 in ACN at 10^{-4} M (**A**) and DBU addition (**B**) in NIR range.

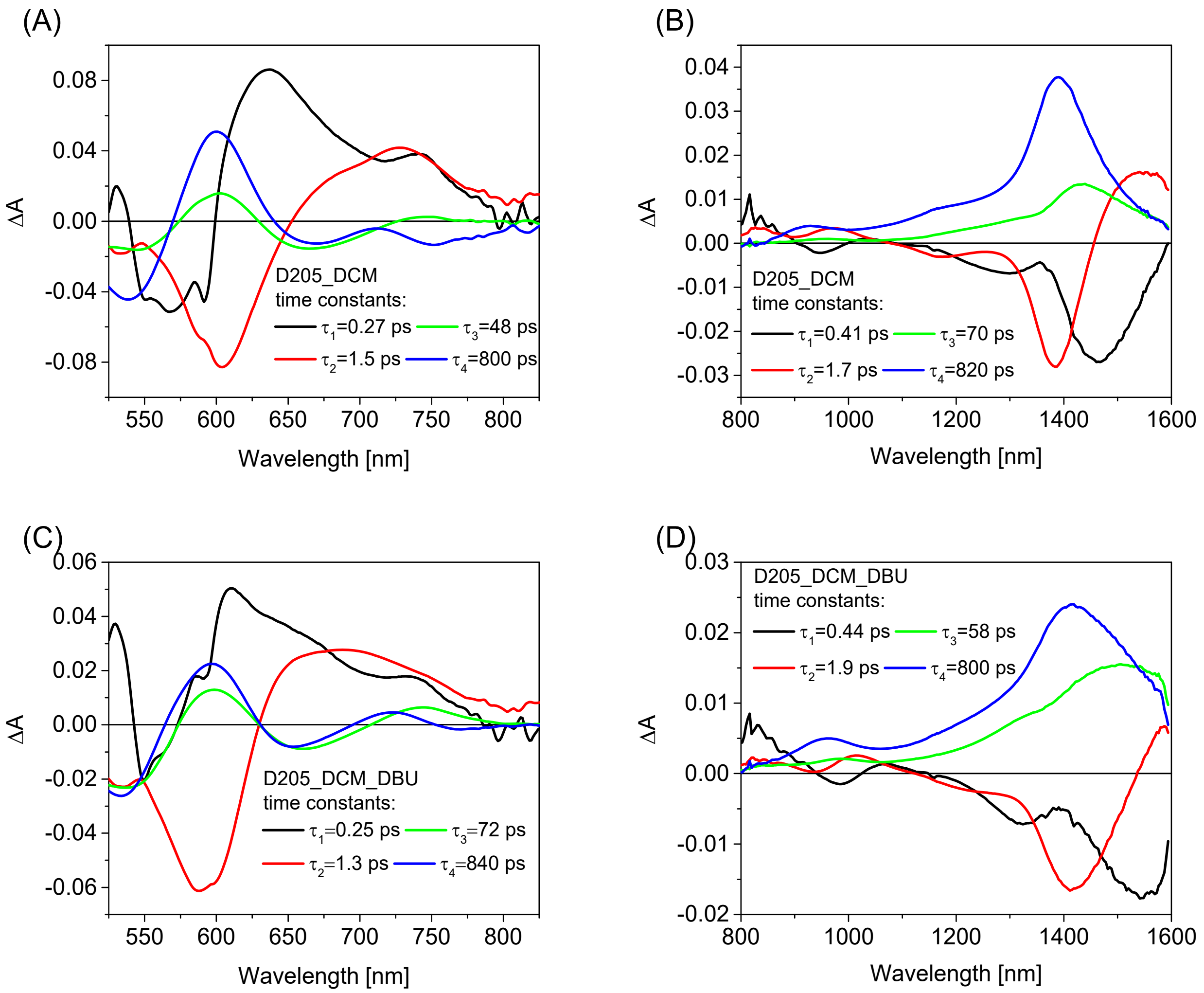

Figure S11. The wavelength-dependent amplitudes of the fitted components (4-exponential fit) and the corresponding time constants of the D205 in DCM and after DBU addition (**A,C:** VIS, **B,D:** NIR).

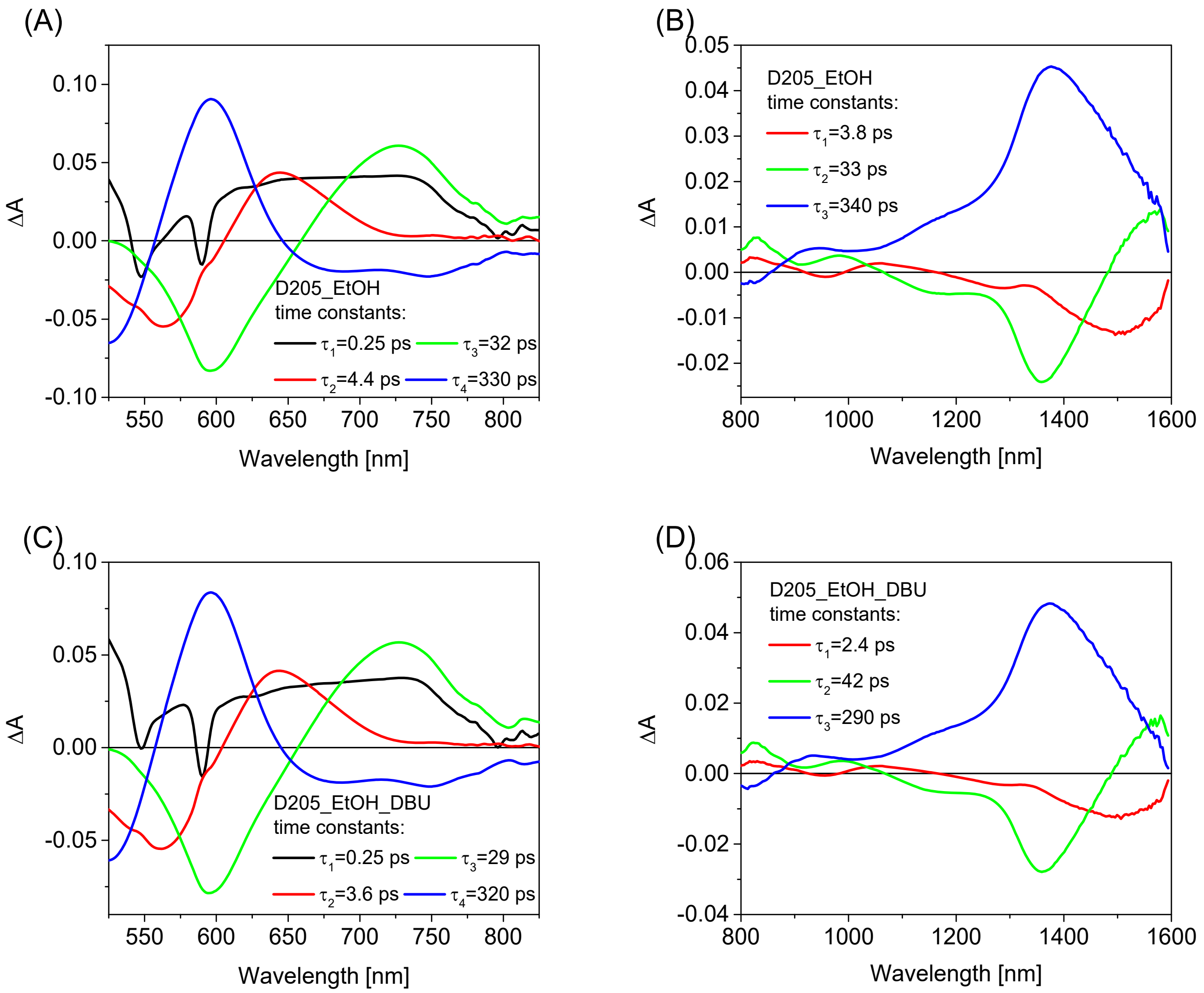

Figure S12. The wavelength-dependent amplitudes of the fitted components (4-exponential fit) and the corresponding time constants of the D205 in EtOH and after DBU addition (**A,C**: VIS, **B,D**: NIR).

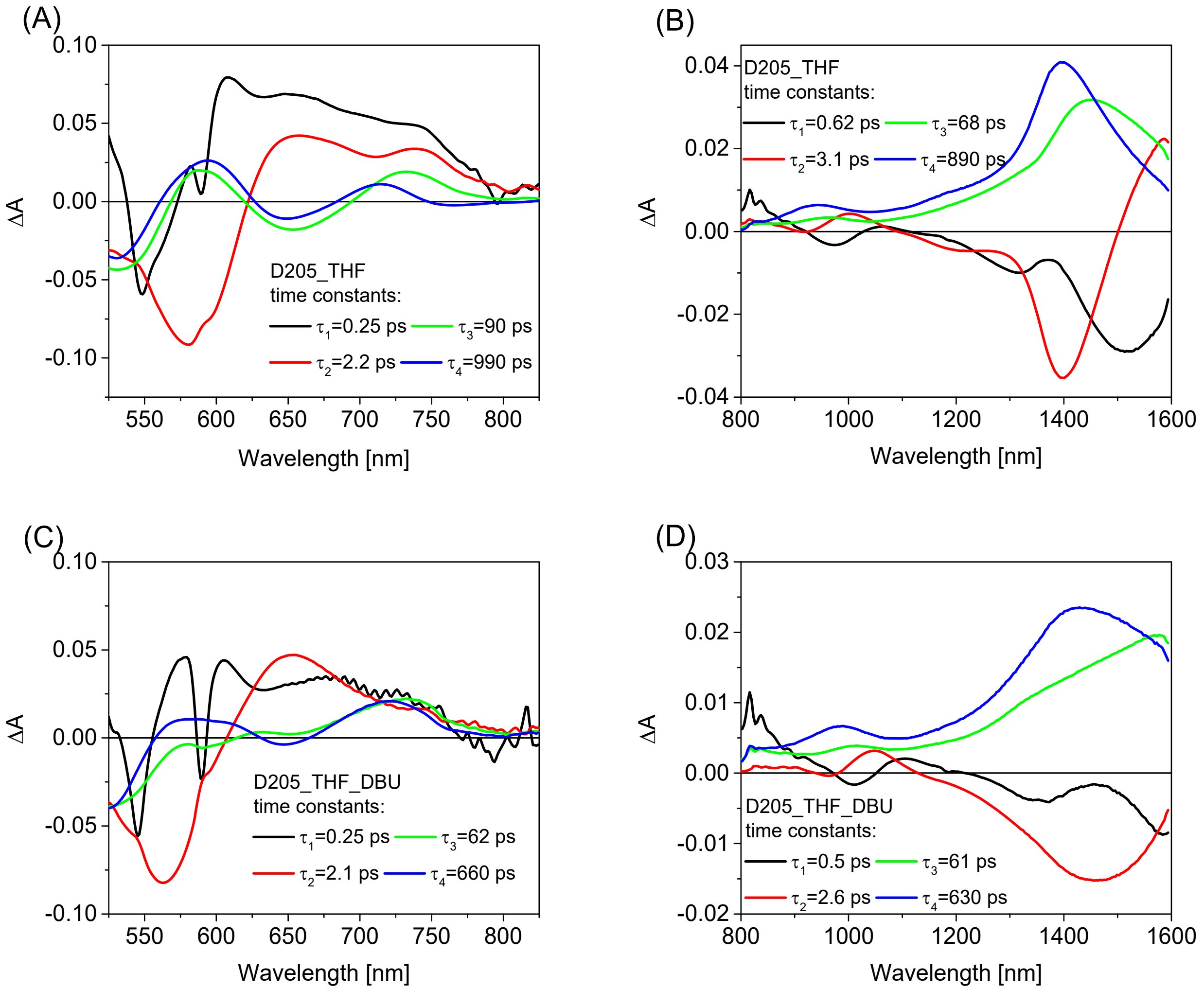

Figure S13. The wavelength-dependent amplitudes of the fitted components and the corresponding time constants of the D205 in THF and after DBU addition (**A,C**: VIS, **B,D**: NIR).

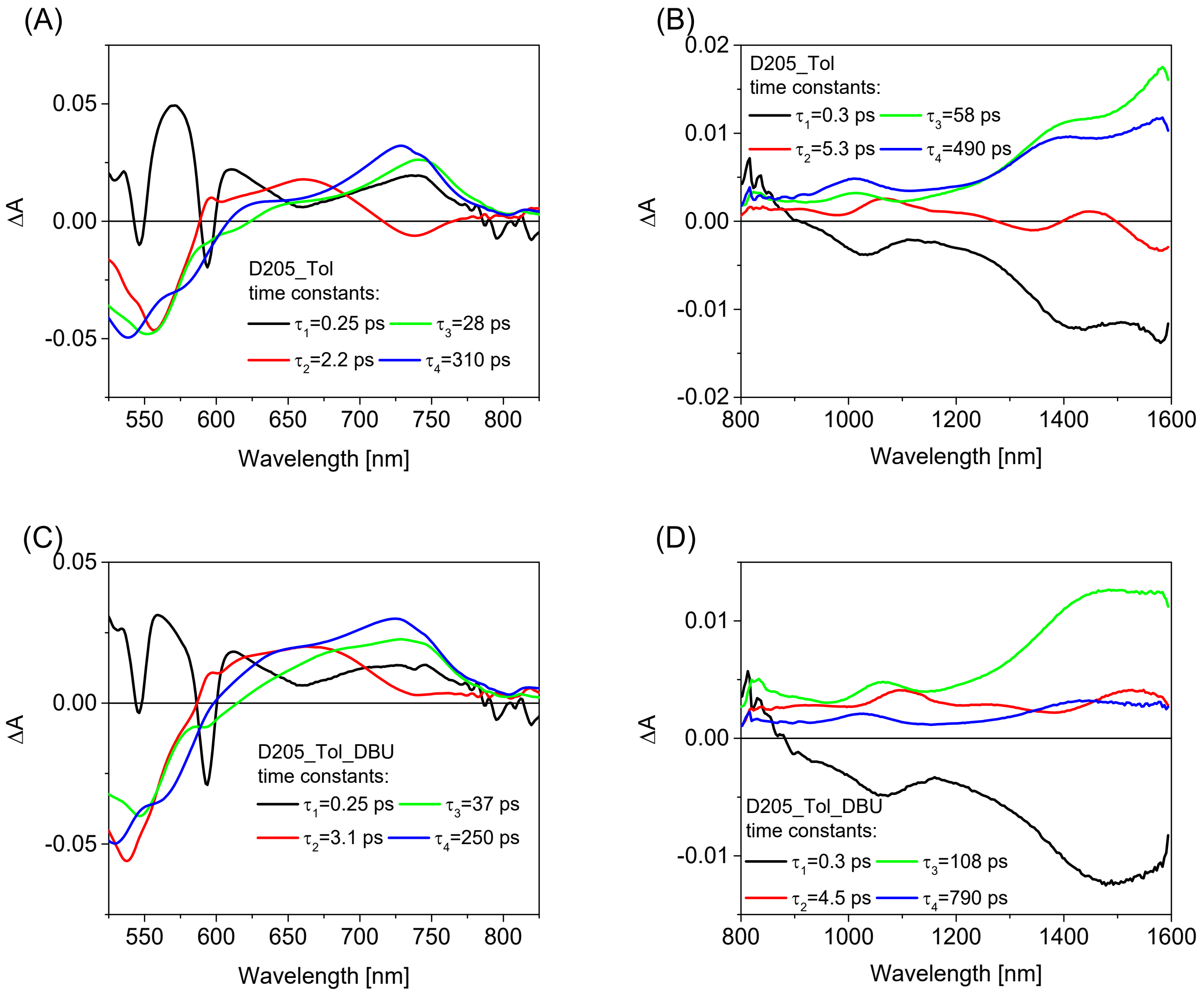

Figure S14. The wavelength-dependent amplitudes of the fitted components (4-exponential fit with the fitted fastest component) and the corresponding time constants of the D205 in Tol and after DBU addition (**A,C**: VIS, **B,D**: NIR).

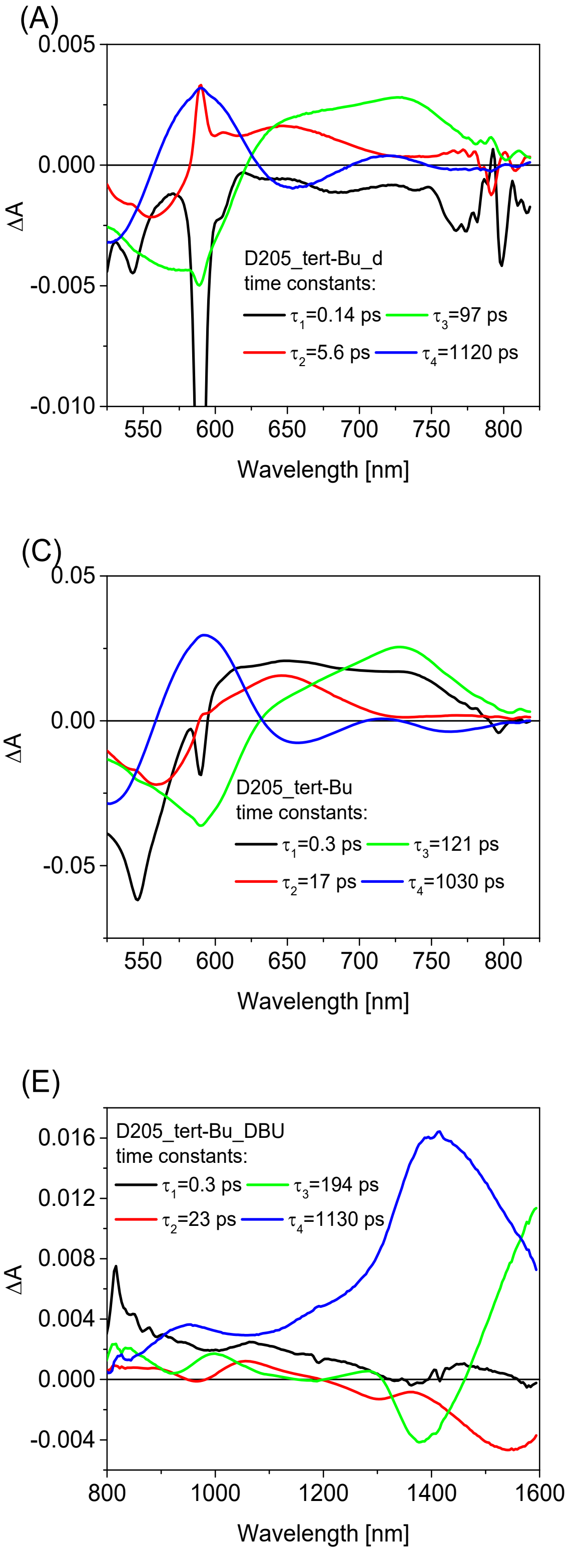

Figure S15. The wavelength-dependent amplitudes of the fitted components (4-exponential fit with the fitted fastest component) and the corresponding time constants of the D205 in tert-Bu at the different dye concentrations and after DBU addition (**A-D**: VIS, **E:** NIR).

Table S7. Theoretical results obtained for D205 and $D205^-$ using the CPCM solvation model with EtOH, ACN, DCM and Tol as solvents. Results for MK2 in a vacuum using different theory levels and the CPCM solvation model with Tol as a solvent (MK2 and $MK2^-$)

**Method**	**HOMO (eV)**	**LUMO (eV)**	**λ (nm)**	***f***	**Coeff**
D205 (EtOH)	-5.144	-2.581	551.6	0.9329	0.70204
$D205^-$ (EtOH)	-5.017	-2.341	530.9	1.0275	0.69899
D205 (ACN)	-5.143	-2.585	551.8	0.9263	0.70216
$D205^-$ (ACN)	-5.038	-2.375	532.5	1.0161	0.69962
D205 (DCM)	-5.148	-2.564	549.3	0.9604	0.70151
$D205^-$ (DCM)	-4.883	-2.147	524.9	0.9686	0.68641
D205 (Tol)	-5.153	-2.494	536.2	1.0269	0.70027
$D205^-$ (Tol)	-4.865	-2.340	514.2	0.0510	0.68355
MK2 in vacuum // Tol (CPCM), neutral forms					
MK2 b3lyp/6-31G(d)	-4.840	-2.528	506.2	1.4239	0.70451
MK2 b3lyp/6-31G(d,p)	-4.842	-2.531	503.8	1.4465	0.70449
MK2 pbe1pbe/6-31G(d)	-5.091	-2.442	544.8	1.4702	0.69827
MK2 cam-b3lyp/6-31G(d)	-6.290	-1.376	388.8	1.5744	0.42957 (H→L), 0.44784 (H-1→L)
MK2 b3lyp/6-31G(d), Tol (CPCM)	-4.865	-2.624	491.1	1.6786	0.70211
$MK2^-$ b3lyp/6-31G(d), Tol (CPCM)	-3.910	-1.177	431.1	1.0434	0.69078

Table S8. Calculated [b3lyp/6-31G(d)] singlet excitation energies with the corresponding oscillator strengths *f* starting from optimized geometry of the ground states of D205 and MK2 using the CPCM solvation model with ACN, EtOH, DCM (D205) and Tol (D205 and MK2) as solvents.

	D205_ACN		D205_EtOH		D205_DCM		D205_Tol		MK2_Tol	
$S_0 \rightarrow S_i$	$E \times 10^{-3}$ / cm^{-1}	f	$E \times 10^{-3}$ / cm^{-1}	f	$E \times 10^{-3}$ / cm^{-1}	f	$E \times 10^{-3}$ / cm^{-1}	f	$E \times 10^{-3}$ / cm^{-1}	f
$\rightarrow S_1$	18.1	0.9263	18.1	0.9329	18.2	0.9604	18.7	1.0269	20.4	1.6786
$\rightarrow S_2$	23.1	0.4984	23.1	0.4982	23.2	0.4963	23.3	0.4744	21.0†	0.0009
$\rightarrow S_3$	24.8	0.362	24.8	0.3662	24.9	0.3823	24.4†	0.0006	22.2	0.2822
$\rightarrow S_4$	25.3†	0.0008	25.2†	0.0007	25.0†	0.0006	25.4	0.4277	24.7†	0.0006
$\rightarrow S_5$	26.7	0.5710	26.6	0.5699	26.6	0.5626	26.7	0.5141	26.4	0.0180
$\rightarrow S_6$	27.9	0.0733	28.0	0.0731	28.0	0.0694	28.2	0.0402	27.1	0.0134
$\rightarrow S_7$	30.0†	0.0003	30.0†	0.0009	30.0†	0.0009	30.2	0.0064	27.9	0.0550
$\rightarrow S_8$	31.3	0.0279	31.3	0.0276	31.4	0.0253	31.5†	0.0006	28.2	0.0239
$\rightarrow S_9$	31.7	0.0059	31.7†	0.0005	31.8	0.0111	31.6	0.0146	30.3	0.0890
$\rightarrow S_{10}$	31.8†	0.0005	31.8†	0.0016	31.9†	0.0003	32.2	0.1252	31.0	0.0265
$\rightarrow S_{11}$	32.5	0.0728	32.5	0.0730	32.3†	0.0001	32.4	0.0052	31.1	0.0218
$\rightarrow S_{12}$	32.6†	0.0002	32.6	0.0187	32.5	0.1367	32.4	0.0926	31.3	0.0519
$\rightarrow S_{13}$	32.6	0.0031	32.6	0.0176	32.6†	0.0001	32.8	0.0138	31.5	0.0303
$\rightarrow S_{14}$	32.7	0.1577	32.7	0.1493	32.7	0.0961	33.0	0.0111	31.7	0.0373
$\rightarrow S_{15}$	33.0	0.0313	33.0	0.0278	33.0	0.0078	33.1†	0.0001	32.2	0.0825

† n,π^* transition, otherwise π,π^*.

Table S9. Calculated [b3lyp/6-31G(d)] singlet excitation energies with the corresponding oscillator strengths *f* starting from optimized geometry of the ground states of $D205^-$ and $MK2^-$ using the CPCM solvation model with ACN, EtOH, DCM (D205) and Tol (D205 and MK2) as solvents.

$S_0 \rightarrow S_i$	D205⁻_ACN		D205⁻_EtOH		D205⁻_DCM		D205⁻_Tol		MK2⁻_Tol	
	$E \times 10^{-3}$ / cm^{-1}	*f*	$E \times 10^{-3}$ / cm^{-1}	*f*	$E \times 10^{-3}$ / cm^{-1}	*f*	$E \times 10^{-3}$ / cm^{-1}	*f*	$E \times 10^{-3}$ / cm^{-1}	*f*
$\rightarrow S_1$	18.8	1.0161	18.8	1.0275	19.1	0.9686	19.4	0.0510	23.2	1.0434
$\rightarrow S_2$	21.4	0.0325	21.2	0.0269	20.1	0.0379	20.6	1.2376	23.5†	0.0007
$\rightarrow S_3$	21.8	0.0523	21.6	0.0540	20.6	0.1573	21.7	0.0304	23.7	0.6127
$\rightarrow S_4$	24.1	0.3693	24.1	0.3564	23.3	0.0109	22.6	0.0110	23.9†	0.0002
$\rightarrow S_5$	24.9	0.0193	24.7	0.0305	24.3	0.3331	24.4	0.0217	25.2	0.0052
$\rightarrow S_6$	25.5	0.5701	25.6	0.5965	25.4†	0.0008	24.8	0.0709	25.3	0.0354
$\rightarrow S_7$	25.7†	0.0002	25.7	0.0094	25.6	0.7369	24.9†	0.0001	26.1	0.0745
$\rightarrow S_8$	26.9	0.3289	27.0	0.2937	27.0	0.1126	25.0	0.0284	26.8	0.0392
$\rightarrow S_9$	28.3	0.0132	28.4	0.0167	27.6†	0.0005	25.1	0.6790	27.2	0.0046
$\rightarrow S_{10}$	28.8	0.0048	28.7	0.0055	28.0	0.0063	25.2	0.0401	28.1†	0.0004
$\rightarrow S_{11}$	29.3†	0.0009	29.1†	0.0009	28.6	0.0798	27.4†	0.0005	28.3†	0.0008
$\rightarrow S_{12}$	31.2	0.1092	31.1	0.1196	30.0	0.0391	28.7	0.0337	28.5	0.0162
$\rightarrow S_{13}$	31.6	0.0259	31.5	0.0144	30.7	0.0324	28.7	0.2029	29.9	0.0124
$\rightarrow S_{14}$	31.9	0.0318	31.9	0.0183	30.8†	0.0004	31.0†	0.0002	30.2	0.0111
$\rightarrow S_{15}$	32.3	0.0055	32.2†	0.0007	31.3	0.039	31.7	0.0514	30.2	0.0278

† n,π^* transition, otherwise π,π^*.

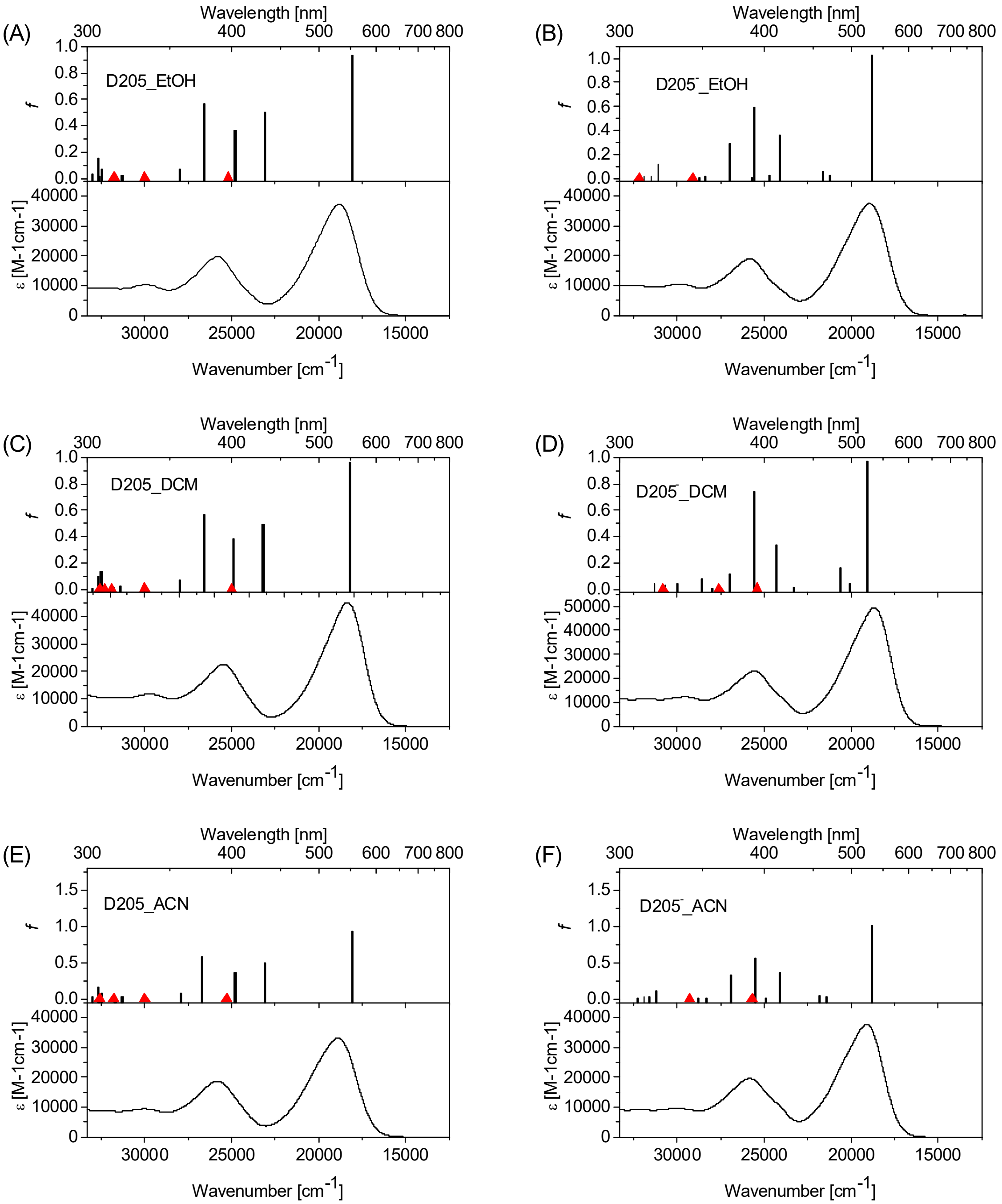

Figure S16. Absorption spectra of D205 (neutral; A, C, E) and D205$^-$ (anion; B, D, F) in EtOH (A,B), DCM (C,D) and ACN (E,F). Predicted transition energies and oscillator strengths *f* are indicated by solid vertical lines. The (prohibited) transitions involving the n,π* singlet stares are marked by red triangles. All theoretical results were determined at the b3lyp/6-31G(d) level of theory.

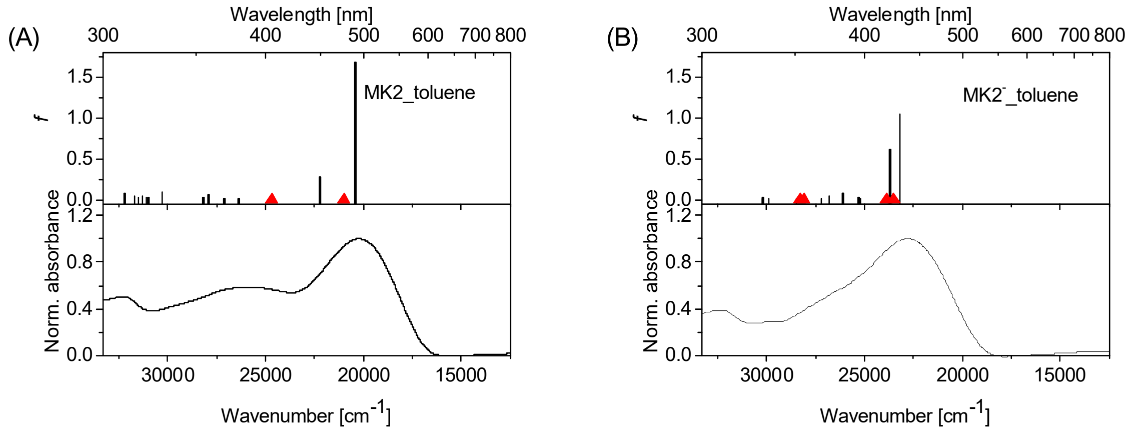

Figure S17. Absorption spectra of MK2 (neutral; A) and $MK2^-$ (anion; B) in Tol. Predicted transition energies and oscillator strengths *f* are indicated by solid vertical lines. The (prohibited) transitions involving the n,π* singlet stares are marked by red triangles. All theoretical results were determined at the b3lyp/6-31G(d) level of theory.

Dye	States	Dominate transitions	FMOs		
D205_ACN	H→L	ICT + (π,π*)	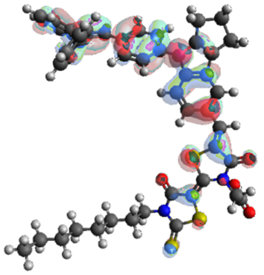	→	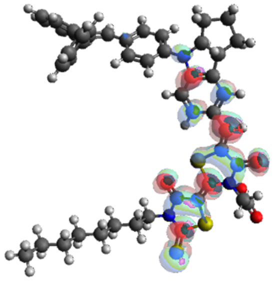
	H-1→L	ICT + (π,π*)	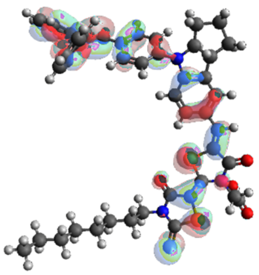	→	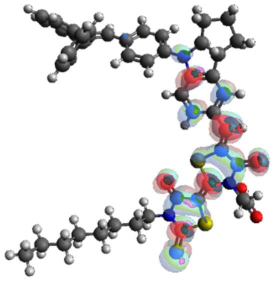
	H→L+1	ICT + (π,π*)	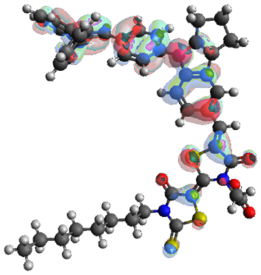	→	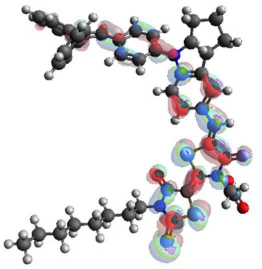
	H-2→L	ICT + (π,π*)	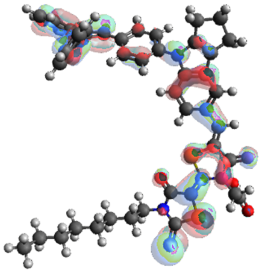	→	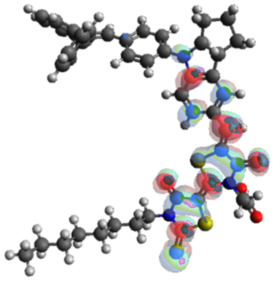
	H-3→L	ICT + (n,π*)	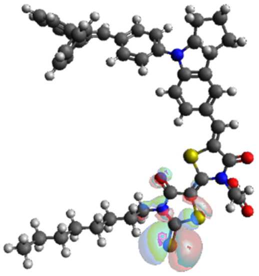	→	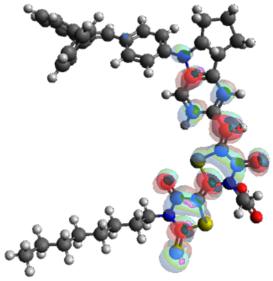

	H^{-3}→L^{+1}	ICT + (n,π*)	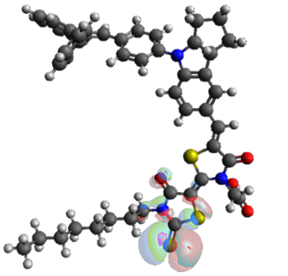	→	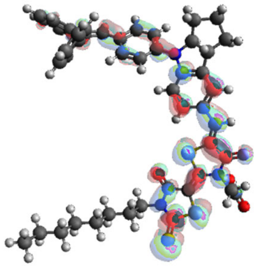
$D205^{-}$ _ACN	H→L	ICT + (π,π*)	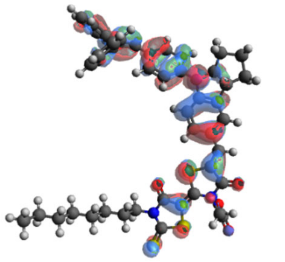	→	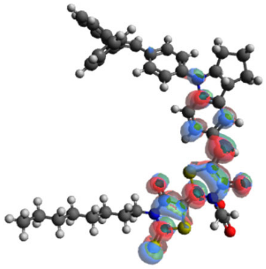

Figure S18. The shape of the HOMO and the LUMO of D205_ACN and $D205^{-}$_ACN at the b3lyp/6-31G(d) theory level responsible for the S_0→S_1 electronic transition determined as isodensity surface plots. The H^{-n}, L^{+m} (π,π*) and (n,π*) are representatives of the nth, mth HOMO and LUMO states, respectively (for D205_ACN).

Dye	States	Dominate transitions	FMOs		
MK2_Tol	H→L	LE + (π,π*)	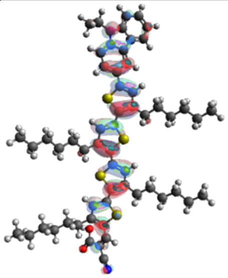	→	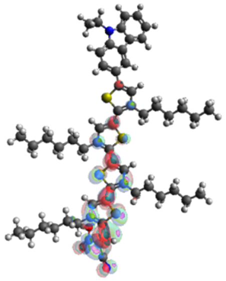
	H^{-1}→L	LE + (n,π*)	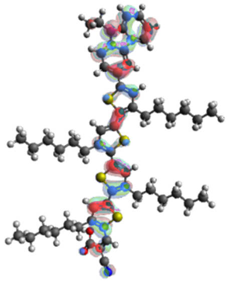	→	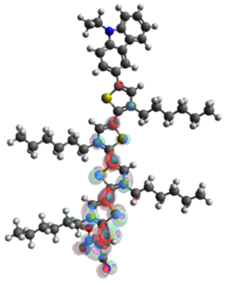
	H→L^{+1}	LE + (n,π*)	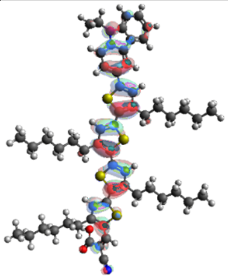	→	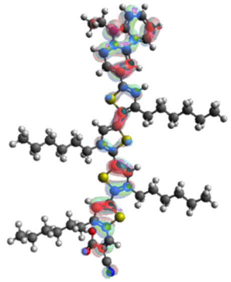
	H^{-2}→L	LE + (π,π*)	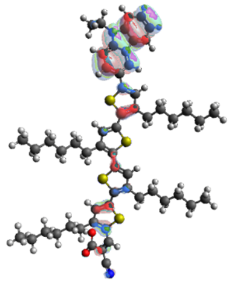	→	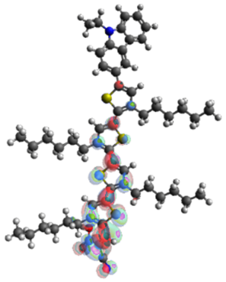
$MK2^{-}$_Tol	H→L	LE + (π,π*)	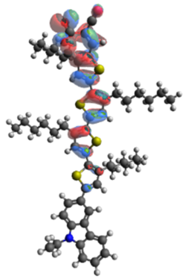	→	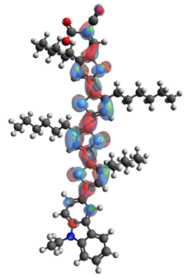

Figure S19. The shape of the HOMO and the LUMO of MK2_Tol and $MK2^{-}$_Tol at the b3lyp/6-31G(d) theory level responsible for the S_0→S_1 electronic transition determined as isodensity surface plots. The H^{-n}, L^{+m} (π,π*) and (n,π*) are representatives of the nth, mth HOMO and LUMO states, respectively (for MK2_Tol).

DSSC characterization:

A UV-VIS-NIR JASCO V-770 spectrophotometer equipped with a 150 mm integrating sphere (LN-925) was used to measure the stationary absorption spectra of photoanodes, after sensitization by MK2 and/or D205 dyes, in the UV-VIS range. The samples were mounted in front of the integrating sphere in order to detect both transmitted and scattered light. Absorption spectra were recorded in the spectral range from 350 nm to 850 nm with a UV-VIS bandwidth of 1 nm. The baselines for the unsensitized TiO_2 electrodes were measured and subtracted from the absorption spectra of the photoanodes sensitized by the dyes. Current–voltage measurements and IPCE (incident photon to current efficiency) spectra of the studied solar cells were recorded using a potentiostat (model M101 with an FRA32M frequency response analyzer module, Autolab) coupled to a photoelectric spectrometer, equipped with a solar simulator (Photon Institute, Poland). Current–voltage measurements were made one hour after cell preparation completion. The photovoltaic parameters presented in the tables are usually averaged over 3 or more samples of the same series. For a few configurations only two cells in the series were prepared, and the parameters for the better cell are shown in the indicated tables. A Xe lamp with an AM 1.5 G spectral filter and irradiance adjusted to 100 mW/cm^2 using a calibrated cell (15151, ABET) mimicked sunlight conditions. Corrected photocurrent efficiency (TotalAPCE) was calculated as the short-circuit current per the number of absorbed photons. This parameter is used to compare the charge transfer efficiency for the cells of different dye absorptions. The complete solar cells with thin TiO_2 layers were used for **transient absorption studies**. The transient absorption system has been described previously by us in more detail (Helios spectrometer, Ultrafast Systems, and Spectra Physics laser system). The laser pulses were set up at 470 nm, and the IRF (pump-probe cross correlation function) duration was about 200 fs (FWHM). The pump pulse energy was set to 150 nJ, which corresponds to an energy density of 75 μJ cm^{-1}. The transient absorption measurements were analyzed using the Surface Explorer software (Ultrafast Systems), including global analysis. The program allowed fitting a multi-exponential function (convoluted with IRF) to the kinetic vectors of a selected number of singular values. Finally, the characteristic time constants and the wavelength-dependent amplitudes associated with them were obtained.

The stationary absorption spectra of MK2 and D205 dyes in solution were measured using the same spectrophotometer (UV-VIS-NIR JASCO V-770) that was used to measure the spectra of photoanodes. In this case, an attachment was applied to measure absorption spectra in solutions. **Femtosecond transient absorption** measurements for D205 and MK2 solutions were made on the same Helios spectrometer, Ultrafast Systems, and Spectra Physics laser system which were used to measure the DSSCs. The laser pulses were set up at 505 nm, and the IRF (pump-probe cross correlation function) duration was about 200 fs (FWHM). The pump pulse energy was set to 1000 nJ, which corresponds to an energy density of 500 μJ cm^{-1}.
